# Characterization of Muscle Tissue Cell Diversity and Clinical Implications in Idiopathic Inflammatory Myopathy

**DOI:** 10.1002/jcsm.70043

**Published:** 2025-08-28

**Authors:** Honglin Zhu, Yizhi Xiao, Shasha Xie, Qiming Meng, Ting Ding, Ting Huang, Di Liu, Sijia Liu, Xiaoli Zhang, Huali Zhang, Hui Luo

**Affiliations:** ^1^ Department of Rheumatology and Immunology, Xiangya Hospital Central South University Changsha Hunan China; ^2^ National Clinical Research Center for Geriatric Disorders, Xiangya Hospital Central South University Changsha Hunan China; ^3^ Provincial Clinical Research Center for Rheumatic and Immunologic Diseases Xiangya Hospital Changsha Hunan China; ^4^ Department of Pathophysiology, Xiangya School of Medicine Central South University Changsha Hunan China

**Keywords:** cellular heterogeneity, disease outcome, idiopathic inflammatory myopathies, scRNA sequencing

## Abstract

**Background:**

Idiopathic inflammatory myopathies (IIMs) exhibit diverse cellular microenvironments in muscle tissues, yet the full spectrum of cell populations and changes remains unclear. This study aimed to characterize cellular heterogeneity, explore cell–cell interactions and assess the prognostic value of cell subtype abundances across IIM subtypes in Han Chinese.

**Methods:**

Muscle samples from six IIMs and three normal controls (NC) underwent single‐cell RNA sequencing (scRNA‐seq), whereas bulk RNA sequencing was performed on 203 IIMs and 19 NC. To avoid potential biases in cell proportion data from scRNA‐seq, we used CIBERSORTx, a robust deconvolution method, to estimate cell subtype abundances in the large IIMs cohort. Cell–cell interaction, correlation and survival analysis were performed to investigate associations between cell subtypes, clinical features and disease progression.

**Results:**

We identified 10 T/NK cell types, eight monocyte/macrophage/dendritic cell types, 10 vascular‐related cell types and four skeletal muscle cell types in IIM muscle tissues, with varying abundances across subgroups. Increased ISG^hi^ T cells (1.42% vs. 0.075% in NC) and ISG^hi^ monocytes (4.24% vs. 0% in NC) in dermatomyositis (DM), particularly in anti‐MDA5 and anti‐NXP2 patients, correlated with skin rashes and higher relapse rates. CD56^dim^CD16^dim^NK cells, exhibiting the highest cytotoxicity, were elevated most in anti‐SRP (11.93% vs. 8.15% in NC) immune‐mediated necrotizing myopathy (IMNM) and associated with severe muscle damage (*p* = 0.0001, rho = 0.267). Reduced angiogenesis‐related SERPINB2^+^ monocytes (37.12% vs. 46.69% in NC) predicted better outcomes in IMNM (*p* = 0.006, HR = 0.264), whereas decreased HIF3A^+^CECs (14.29% in DM vs. 16.95% in NC), essential for endothelial barrier maintenance, negatively correlated with myofiber necrosis (*p* = 0.016, rho = −0.168) and were predictive of improved outcomes in DM (*p* = 0.014, HR = 0.412). Elevated endothelial‐like pericytes in antisynthetase syndrome (ASS, 55.34% vs. 50.02% in NC) and IMNM (54.42%) were linked to muscle damage (*p* < 0.0001, rho = 0.272). Certain key pathways, such as angiogenesis‐related pathways, were linked to better outcomes in DM (*p* = 0.002, HR = 0.405), whereas increased cytotoxicity scores, cell chemotaxis and regulation of inflammatory response were associated with a higher risk of relapse in both DM and IMNM. We also observed a reduction in Type I muscle fibres (22.66% in ASS vs. 66.68% in NC) that express MIF and MHC class I molecules and show extensive interactions with inflammatory cells via MIF‐CD74 ligand–receptor signalling.

**Conclusions:**

Our findings reveal significant shifts in cell subpopulations within IIM muscle tissues, which may contribute to muscle damage and influence disease outcomes.

## 1 Background

Idiopathic inflammatory myopathies (IIMs) encompass a group of highly diverse systemic autoimmune disorders characterized by chronic muscle weakness, inflammation and extramuscular symptoms [1]. These disorders are further categorized into several subtypes: dermatomyositis (DM), juvenile dermatomyositis (JDM), anti‐synthetase syndrome (ASS), immune‐mediated necrotizing myopathy (IMNM), polymyositis (PM), inclusion body myositis (IBM) and overlap myositis. However, certain clinical or histopathological features are shared among several of these subtypes. To distinguish between the subtypes of IIMs with more uniform clinical characteristics, myositis‐specific autoantibodies (MSAs) have become increasingly utilized in recent years. Typically, autoantibodies targeting MDA5, Mi‐2, SAE, NXP2 and TIF1γ are associated with DM; autoantibodies against tRNA synthetases (such as Jo1, PL‐7, PL‐12 and EJ) are linked to ASS; and autoantibodies targeting SRP and HMGCR are identified in IMNM [1].

Muscle pathology in IIMs is marked by infiltrates of various T cells, B cells, plasma cells and plasmacytoid dendritic cell phenotypes [2, 3, 4], with effector and tissue‐resident memory T cells linked to disease progression [5]. Additionally, endothelial cell loss and dysfunction are observed in JDM and DM, and impaired vascular regeneration has been noted in anti‐Jo1 ASS and IMNM [6, 7, 8]. Endothelial biomarkers of activation and dysfunction can reflect clinical heterogeneity and help predict treatment response in JDM [9]. Understanding these diverse cellular microenvironments in muscle tissue is crucial for clarifying the aetiology, pathogenic mechanisms and treatment responses of IIMs.

However, the spectrum of cell populations and their changes in muscle tissues remains poorly understood, and the interactions between inflammatory cells and muscle fibres have yet to be systematically investigated across IIM subtypes. The prevalence and severity of muscle involvement in MDA5‐positive DM vary among studies [10]. Intriguingly, IBM is exceedingly rare in China, accounting for only 0.68% of neuromuscular disorders, in stark contrast to the United States, where it is the most common form of IIMs in older adults [11]. This disparity suggests that ethnicity may play a role in its prevalence and that the mechanisms underlying muscle damage in IIMs may differ across populations.

In this study, we investigated the cellular heterogeneity and functional states of muscle tissues across IIM subtypes in the Han Chinese population using single‐cell RNA sequencing (scRNA‐seq), emphasizing the interactions between skeletal muscle cells, immune cells and endothelial cells. Utilizing single‐cell signatures, we deconvoluted bulk RNA‐seq data from a large cohort of 203 IIMs to estimate the proportions of cell subtypes in muscle tissue. Additionally, we analysed the correlations between these cell subtype proportions, clinical indices and tissue pathology. Finally, we evaluated the potential of these cell subtype proportions as prognostic markers for IIMs.

## 2 Materials and Methods

### 2.1 Subjects and Skeletal Muscle Biopsies

This study enrolled 209 newly diagnosed IIM patients and 22 normal controls (NC) from the Department of Rheumatology and Immunology at Xiangya Hospital. Patients were classified as DM (according to the ENMC 2018 DM criteria [12]), ASS (autoantibodies against aminoacyl tRNA synthetases, Connors' ASS criteria and 273rd ENMC ASS workshop [13, 14]) or IMNM (ENMC 2016 criteria [15]). Based on MSAs, patients were further grouped into anti‐MDA5, anti‐Mi‐2, anti‐SAE, anti‐NXP2, anti‐TIF1γ, anti‐Jo1, anti‐EJ, anti‐PL‐7, anti‐PL‐12, anti‐SRP, anti‐HMGCR and nonautoantibody subgroups. MSAs were detected using an 18‐antibody kit (Euroimmun, Germany). Only IIM patients with a single strong positive autoantibody (++) or (+++) were included. Exclusion criteria included infections, tumours, overlap syndromes, severe cardiovascular/metabolic disease and prior use of immunosuppressants.

For scRNA‐seq, six IIM samples and three NC samples were used, including DM (three anti‐MDA5), ASS (two anti‐EJ and one anti‐PL‐12) and NC (three samples). For bulk RNA‐seq, 203 IIMs and 19 NC samples were analysed. The IIM patients were classified into DM (*n* = 121), ASS (*n* = 45) and IMNM (*n* = 37) groups and further divided into subgroups by autoantibodies: anti‐MDA5 (*n* = 57), anti‐Mi‐2 (*n* = 10), anti‐SAE (*n* = 6), anti‐NXP2 (*n* = 8), anti‐TIF1γ (*n* = 20), anti‐Jo1 (*n* = 25), anti‐EJ (*n* = 9), anti‐PL‐7 (*n* = 7), anti‐PL‐12 (*n* = 4), anti‐SRP (*n* = 20), anti‐HMGCR (*n* = 8) and no autoantibodies (*n* = 29) (Table S1).

All IIM patients exhibited proximal upper extremity weakness, assessed by the Manual Muscle Test‐8 (MMT‐8). A skeletal muscle biopsy was performed on the medial aspect of the left biceps brachii for diagnostic purposes. Myogenic damage in the right biceps brachii was detected via electroneuromyography. NC biopsies were obtained from subjects undergoing shoulder or knee arthroplasty (14 from the biceps brachii and eight from the quadriceps femoris). For scRNA‐seq, NC samples were taken from the biceps brachii.

All IIMs and NC participants were Han Chinese. Informed consent was obtained from all participants, and the ethics committee approved the study at Xiangya Hospital, Central South University (Changsha, Hunan, China, ID: 201303293). Patients or the public were not involved in this research's design, conduct, reporting or dissemination plans.

### 2.2 Statistical Analysis

Data normality was assessed using the Shapiro–Wilk test. For continuous data, between‐group comparisons were performed using an unpaired *t*‐test (for normally distributed samples) or the Wilcoxon test (for nonnormally distributed samples). When multiple *t*‐tests or Wilcoxon tests were conducted, false discovery rate (FDR) correction was applied to adjust the *p* value. Comparisons of multiple proportions were conducted using Fisher's exact test. Spearman's rank correlation coefficients were used for correlations between two continuous variables, whereas point‐biserial correlation coefficients were used for correlations between continuous and dichotomous variables. All analyses were performed in R (v4.0.3) using the MVN (v5.8), rstatix (v0.6.0) and correlation (v0.5.0) packages; *p* values of < 0.05 were considered statistically significant, and all tests were two‐sided.

Detailed methods for scRNA‐seq sample preparation and data analysis, RNA extraction and bulk RNA‐seq, CIBERSORTx analysis, Gene Set Enrichment Analysis (GSEA), cell–cell interaction analysis, survival analysis and immunofluorescence staining are provided in the Supporting Information.

## 3 Results

### 3.1 T and NK Cell Subtypes in IIM Muscle Tissues

We performed scRNA‐seq on six newly diagnosed IIM patients (three with DM and three with ASS) and three NC, yielding a total of 34,972 cells after quality control. These cells were well integrated by disease status and sample. Using canonical lineage markers, we identified 11 distinct clusters in muscle tissue: T cells, B cells, NK cells, monocytes (Mo)/macrophages (Mφ), neutrophils, myogenic precursor cells (MPCs), skeletal muscle cells, fibroblasts, smooth muscle cells (SMCs), pericytes (PCs) and endothelial cells (ECs) (Figure S1a,b).

**FIGURE 1:**
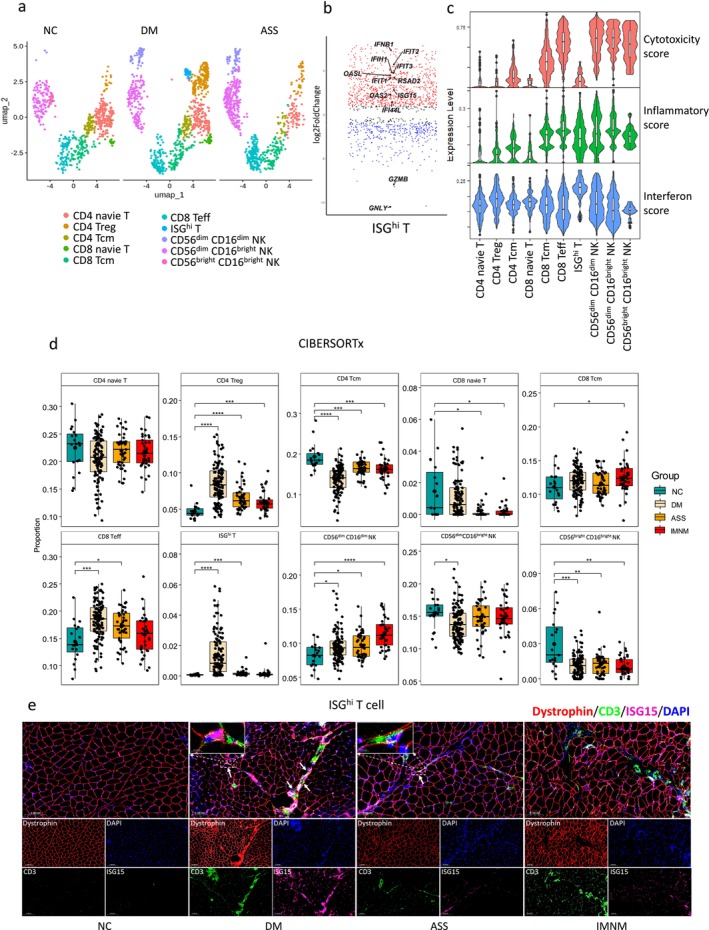
scRNA‐seq analysis of lymphocyte subpopulations in IIM muscle tissues. (a) UMAP plot of combined lymphocyte subpopulations from three DM, three ASS and three NC muscle tissues. (b) Scatter plot showing the expression of selected marker genes in the ISG^hi^ T cell cluster. (c) Violin plots illustrate cytotoxicity, inflammation and interferon scores in lymphocyte subpopulations. (d) Relative proportions of individual lymphocyte subpopulations in the muscle tissues of IIM main subtypes and NC as determined by CIBERSORTx analysis. *p < 0.05, **p < 0.01, ***p < 0.001, ****p < 0.0001 by unpaired *t*‐test (for normally distributed data) or Wilcoxon test (for nonnormally distributed data). (e) Immunofluorescence revealed the distribution and abundance of ISG^hi^ T cells in IIM muscle tissues, stained with dystrophin (red, sarcolemma marker), CD3 (green, T cell marker), ISG15 (purple) and DAPI (blue, nuclei).

We identified 10 T and NK cell subpopulations, which were classified based on marker genes as CD4 naive T, CD4 Treg, CD4 Tcm, CD8 naive T, CD8 Tcm, CD8 Teff, ISG^hi^ T, CD56^dim^CD16^dim^ NK, CD56^dim^CD16^bright^ NK and CD56^bright^CD16^bright^ NK cells (Figures 1a and S2a). Notably, ISG^hi^ T cells exhibited high expression of interferon‐stimulated genes (ISGs) such as *IFIT1*, *IFIT2*, *RSAD2* and *ISG15*, with very low expression of cytotoxic molecules *GZMB* and *GNLY* (Figure 1b). Within this ISG^hi^ T cell population, 7.5% were CD4 positive, 73.6% were CD8 positive, and 18.9% were double‐negative for CD4 and CD8 (Figure S2b). Gene Ontology (GO) enrichment analysis indicated significant enrichment of pathways related to interferon signalling and production, lymphocyte differentiation, migration and proliferation in ISG^hi^ T cells (Figure S2c). We evaluated the functional phenotypes of each T and NK subset by analysing cytotoxicity, inflammatory and interferon signature scores. CD8 Teff and all three NK subtypes exhibited the highest cytotoxicity and inflammatory scores, whereas ISG^hi^ T cells displayed significantly elevated interferon scores in DM (Figures 1c and S2d).

To address potential biases in cell fractions identified by scRNA‐seq, such as variability in cell capture during mechanical dissociation or enzymatic digestion, recovery rates and the small sample size [16, 17], we used CIBERSORTx to estimate cell proportions in bulk RNA‐seq data from muscle tissues of 203 IIM patients. CIBERSORTx, a widely used deconvolution method, performs ‘digital purification’ of transcriptomes from bulk RNA mixtures, enabling the analysis of individual cell types without physical isolation. Often referred to as ‘digital cytometry’ [18, 19, 20, 21], it has been applied to various tissues [22, 23, 24] and in our previous study [25].

We observed shifts in cell type proportions in IIM muscles compared with NC muscles. CD4 Treg and CD56^dim^CD16^dim^ NK cells increased, whereas CD4 Tcm and CD56^bright^CD16^bright^ NK cells decreased in all IIMs. Additionally, ISG^hi^ T cells were significantly expanded in DM (1.42% compared to 0.075% in NC) (Figure 1d). These results were largely consistent with the scRNA‐seq findings, except for CD56^dim^CD16^bright^ NK cells, which were elevated in the ASS group in scRNA‐seq data but unchanged in CIBERSORTx analysis (Figure S2e). GSEA further supported the CIBERSORTx results (Figure S2f). Immunofluorescence confirmed the presence of a subset of ISG^hi^ T cells in DM, with a very small number also observed in ASS. In contrast, these cells were scarcely detected in NC and IMNM samples (Figure 1e). Notably, CD4^+^, CD8^+^ and double‐negative (CD4^−^CD8^−^) ISG^hi^ T cells were all identified in DM muscle tissues (Figure S3a).

In IIMs antibody subtypes, CD4 Treg increased in all IIMs antibody subtypes, and CD4 Tcm decreased in all antibody subtypes except anti‐PL‐12 and anti‐PL‐7. CD8 naive T decreased in anti‐Jo1, CD8 Teff increased in anti‐MDA5, anti‐TIF1γ, anti‐SAE and anti‐Jo1 groups, ISG^hi^ T increased in all DM antibody subgroups (highest in anti‐NXP2 group, 2.47% vs. 0.075% in NC), CD56^dim^CD16^dim^ NK increased most in anti‐SRP group (11.93% compared to 8.15% in NC), CD56^dim^CD16^bright^ NK decreased in anti‐MDA5 and CD56^bright^CD16^bright^ NK decreased most in anti‐SRP group (1.03% compared to 2.97% in NC) (Figure S3b).

In summary, ISG^hi^ T cells are significantly expanded in DM muscle tissues, particularly in the anti‐NXP2 group. CD56^dim^CD16^dim^ NK cells are most elevated in anti‐SRP IMNM, whereas CD56^bright^CD16^bright^ NK cells show the most significant reduction in this group.

### 3.2 Myeloid Cell Subpopulation Analysis in Muscle Tissues of IIMs

**FIGURE 2:**
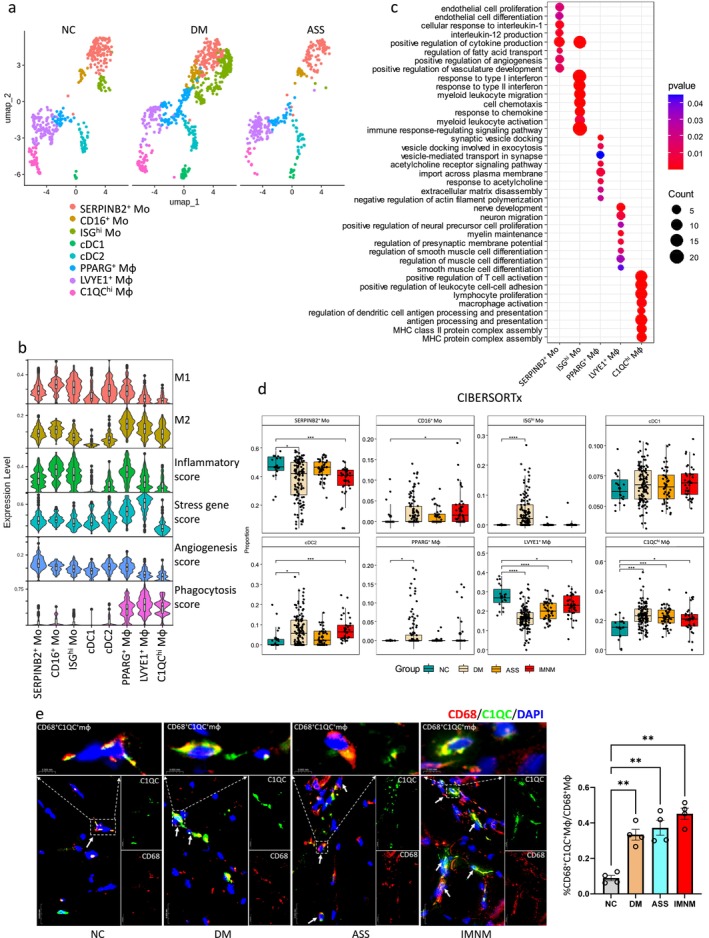
scRNA‐seq profiling of myeloid cell subpopulations in muscle tissues from IIMs. UMAP visualization depicting the integrated myeloid cell subpopulations across three DM, three ASS and three NC muscle tissues. (b) Violin plots illustrating M1, M2, inflammatory, stress, angiogenesis and phagocytosis scores across myeloid cell subpopulations. (c) Functional enrichment analysis using GO terms for top marker genes identified in myeloid cell subpopulations. (d) Comparative analysis of individual myeloid cell subpopulation proportions between IIMs main subtypes and NC using CIBERSORTx. *p < 0.05, **p < 0.01, ***p < 0.001, ****p < 0.0001, determined by unpaired *t*‐test (for normally distributed data) or Wilcoxon test (for nonnormally distributed data). (e) Immunofluorescence identified C1QC^hi^ Mφ in IIM muscle tissues, stained with CD68 (red, Mφ marker), C1QC (green) and DAPI (blue, nuclei). The bar graph shows the proportion of C1QC^hi^ Mφ among total Mφ across IIM subtypes. **p < 0.01 by Wilcoxon test.

Upon reclustering the myeloid cell subpopulation, we identified eight major subpopulations: SERPINB2^+^ Mo, CD16^+^ Mo, ISG^hi^ Mo, conventional dendritic cells (cDC)1, cDC2, PPARG^+^ Mφ, LVYE1^+^ Mφ and C1QC^hi^ Mφ (Figures 2a and S4a). The ‘classically activated’ M1 and ‘alternatively activated’ M2 Mφ polarization system has been used to describe the in vitro activation state of Mφ [26]. Using signature genes of M1/M2 Mφ, we found that PPARG^+^ Mφ exhibited both higher M1 and M2 signatures. Similar to previous studies, LVYE1^+^ Mφ and C1QC^hi^ Mφ exhibited higher M2 signatures (Figure 2b). Functional profiling revealed that CD16^+^ Mo, ISG^hi^ Mo and PPARG^+^ Mφ exhibited elevated inflammatory scores in DM. LYVE1^+^ Mφ showed the highest stress response scores, particularly in ASS, whereas SERPINB2^+^ Mo demonstrated the strongest angiogenic activity in NC. PPARG^+^ Mφ, LYVE1^+^ Mφ and C1QC^hi^ Mφ were associated with phagocytosis across all IIM subtypes (Figures 2b and S4b). GO functional analysis further clarified that SERPINB2^+^ Mo was involved in angiogenesis and cytokine production (e.g., interleukin [IL]‐1 and IL‐12); ISG^hi^ Mo participated in interferon signalling response and cell chemotaxis; PPARG^+^ Mφ was engaged in vesicle docking and transport and the acetylcholine receptor signalling pathway; LVYE1^+^ Mφ contributed to nerve development, myelin maintenance and smooth muscle cell differentiation; C1QC^hi^ Mφ primarily participated in antigen processing and presentation (Figure 2c). Additionally, SERPINB2^+^ Mo, CD16^+^ Mo and ISG^hi^ Mo were linked to endothelial cell proliferation and myeloid leukocyte migration. These subsets and PPARG^+^ Mφ showed heightened cell chemotaxis and myeloid leukocyte activation, particularly in DM. cDC2 were enriched for chemokine‐mediated signalling, with increased activity in both DM and ASS (Figure S4b).

Compositional analysis from CIBERSORTx showed a decrease in LVYE1^+^ Mφ and an increase in C1QC^hi^ Mφ in IIMs. In DM and IMNM, SERPINB2^+^ Mo decreased while cDC2 increased. ISG^hi^ Mo (4.24% vs. 0% in NC) and PPARG^+^ Mφ(1.97% vs. 0% in NC) were elevated in DM (Figure 2d). These changes were consistent across analyses, except for C1QC^hi^ Mφ, which decreased in scRNA‐seq but increased in both the CIBERSORTx and GSEA results (Figure S5a,b). Immunofluorescence confirmed the increase of C1QC^hi^ Mφ in IIM muscle tissues, with the highest levels observed in IMNM (Figure 2e).

SERPINB2^+^ Mo decreased in the anti‐MDA5 (37.27% vs. 46.69% in NC), anti‐TIF1γ (35.50%) and anti‐SRP groups (36.58%). CD16^+^ Mo increased in the anti‐NXP2 group (6.32% vs. 1.29% in NC), whereas ISG^hi^ Mo increased across all DM antibody subgroups, with the highest levels in the anti‐NXP2 group (10.21% vs. 0% in NC). cDC2 increased in the anti‐MDA5 (7.47% vs. 3.19% in NC) and anti‐SRP (7.85%) groups. PPARG^+^ Mφ increased in the anti‐MDA5 (2.73% vs. 0% in NC) and anti‐NXP2 (4.28%) groups. LVYE1^+^ Mφ decreased across all DM antibody subgroups and in the anti‐Jo1 group (18.98% vs. 27.61%). C1QC^hi^ Mφ increased in the anti‐MDA5 (24.78% vs. 14.75% in NC), anti‐TIF1γ (22.21%), anti‐SAE (24.91%), anti‐Mi‐2 (23.26%) and anti‐Jo1 (23.08%) groups (Figure S5c).

Collectively, we observed a more complex macrophage phenotype in vivo. Angiogenesis‐related SERPINB2^+^ Mo and tissue homeostasis‐related LVYE1^+^ Mφ decreased in IIMs, whereas antigen processing and presentation‐related C1QC^hi^ Mφ increased. Notably, ISG^hi^ Mo was expanded in the anti‐NXP2 DM group. Beyond changes in abundance, these cell subtypes also display functional alterations in the context of IIMs.

### 3.3 Vascular‐Related Cell Subpopulation Profiling in Muscle Tissues of IIMs

**FIGURE 3:**
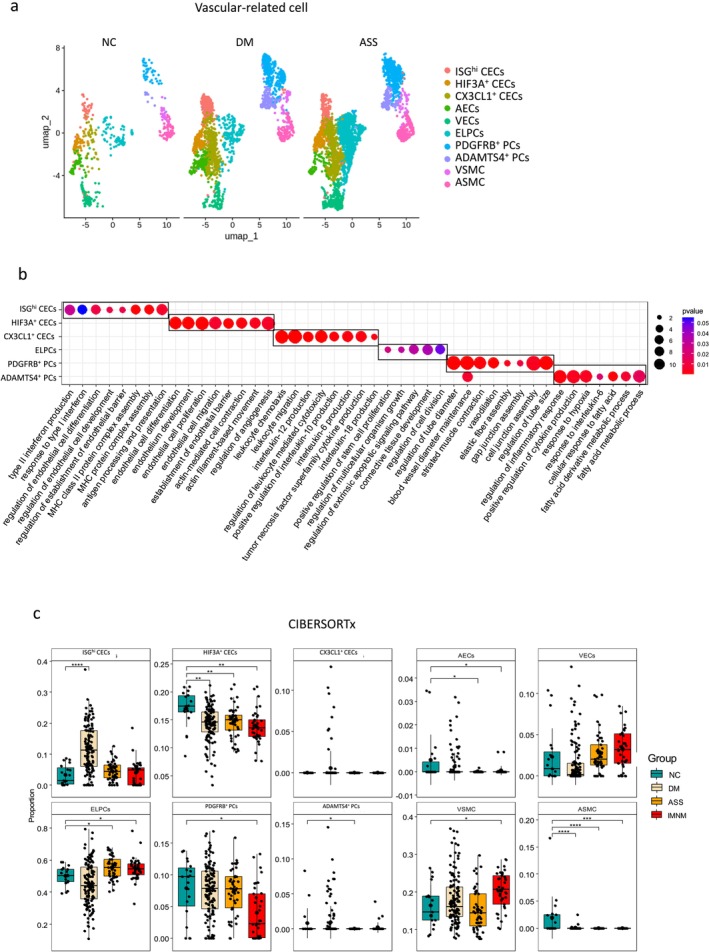
Vascular‐related cell subpopulations in muscle tissues from IIMs. (a) UMAP visualization displaying integrated vascular‐related cell subpopulations across three DM, three ASS and three NC muscle tissues. (b) Functional enrichment analysis using GO terms for top marker genes identified in vascular‐related cell subpopulations. (c) Comparative analysis of individual proportions of vascular‐related cell subpopulations between different IIM subtypes and NC using CIBERSORTx. *p < 0.05, **p < 0.01, ***p < 0.001, ****p < 0.0001, determined by unpaired *t*‐test (for normally distributed data) or Wilcoxon test (for nonnormally distributed data).

Vascular‐related subcluster analysis revealed 10 subpopulations: ISG^hi^ capillary endothelial cells (CECs), HIF3A^+^ CECs, CX3CL1^+^ CECs, arterial endothelial cells (AECs), venous endothelial cells (VECs), endothelial‐like pericytes (ELPCs), PDGFRB^+^ pericytes (PCs), ADAMTS4^+^ PCs, vascular smooth muscle cells (VSMCs) and arterial smooth muscle cells (ASMCs) (Figures 3a and S6a). GO functional analysis indicated that ISG^hi^ CECs are involved in interferon response and MHC protein complex assembly. HIF3A^+^ CECs participate in angiogenesis and the establishment of the endothelial barrier. CX3CL1^+^ CECs are implicated in cytokine production (such as IL‐6, IL‐10 and IL‐12), leukocyte chemotaxis and migration. ELPCs are involved in stem cell proliferation, cell division and connective tissue development. PDGFRB^+^ PCs maintain blood vessel diameter, assemble gap and cell junctions and regulate tube size. ADAMTS4^+^ PCs participate in inflammatory responses and fatty acid metabolic processes (Figure 3b).

To investigate functional differences in myeloid subsets across IIM subtypes, we analysed alterations in key biological pathways. Pathways related to angiogenesis regulation and actin filament bundle assembly—enriched in HIF3A^+^ CECs, CX3CL1^+^ CECs, AECs, VECs and ELPCs—were downregulated in both DM and ASS, with a more pronounced reduction in ASS. In contrast, leukocyte migration pathways were upregulated in these endothelial cell subsets in both DM and ASS. Leukocyte tethering/rolling and chemotaxis‐related pathways were predominantly enriched in VECs. ADAMTS4^+^ PCs were associated with fatty acid metabolism and inflammatory regulation, VSMCs and ASMCs were involved in muscle development pathways, which were suppressed in both DM and ASS (Figure S6b).

Compositional analysis using CIBERSORTx showed an increase in ISG^hi^ CECs in DM (11.96% vs. 3.25% in NC), whereas HIF3A^+^ CECs and ASMC were reduced in IIMs. In ASS and IMNM, AECs decreased, whereas ELPCs increased. PDGFRB^+^ PCs decreased (3.98% vs. 7.85% in NC), and VSMCs were elevated (20.40% vs. 16.41% in NC) in IMNM (Figure 3c). These changes were consistent with scRNA‐seq data, with minor discrepancies observed for PDGFRB^+^ PCs and VSMCs, whereas GSEA results aligned well with the CIBERSORTx findings (Figure S7a,b).

In the IIMs antibody subgroups, ISG^hi^ CECs increased in anti‐MDA5 (16.47% vs. 3.25% in NC), anti‐TIF1γ (10%) and anti‐NXP2 (11.20%) groups, whereas HIF3A^+^ CECs decreased in anti‐MDA5(14.36% vs. 16.95% in NC), anti‐TIF1γ (13.52%) and anti‐Jo1 (14.69%) groups. CX3CL1^+^ CECs exhibited an increase specifically in the anti‐NXP2 (2.18% vs. 0% in NC) group. ELPCs decreased in anti‐MDA5(40.94% vs. 50.02% in NC) and anti‐NXP2 (35.07%) groups, whereas ADAMTS4^+^ PCs increased in the anti‐NXP2 group (4.33% vs. 0.79% in NC). VSMC increased in the anti‐SRP group (21.51% vs. 16.41% in NC), whereas ASMC decreased in the anti‐MDA5 (0.04% vs. 1.91% in NC), anti‐Jo1 (0%) and anti‐SRP groups (0%) (Figure S7c).

Vascular subcluster analysis identified 10 vascular‐related cell subsets with distinct functional profiles. In IIMs, angiogenesis‐related pathways were generally downregulated, particularly in ASS, whereas leukocyte migration was upregulated. Notable shifts included increased ISG^hi^ CECs in DM and antibody subgroups and decreased HIF3A^+^ CECs and ASMC in all IIMs. AECs, ELPCs and VSMCs showed subtype‐ and antibody‐specific alterations.

### 3.4 Skeletal Muscle Cell Subpopulations and Their Interactions With the Immune Microenvironment

**FIGURE 4:**
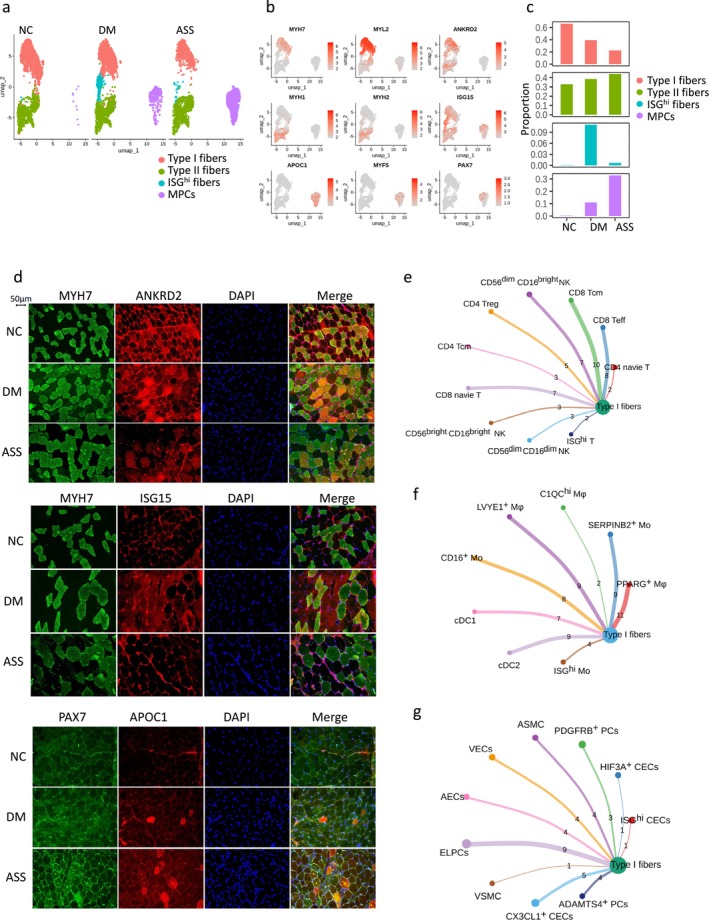
scRNA‐seq analysis of IIMs skeletal muscle cell subpopulations. (a) UMAP plot showing skeletal muscle cell subpopulations from three DM, three ASS and three NC muscle tissue samples. (b) Feature plot depicting the expression of selected marker genes in each cluster, highlighting the expression of myosin heavy chain (*MYH*) and myosin light chain (*MYL*) genes to distinguish ‘slow‐twitch’ (Type 1, *MYH7*) and ‘fast‐twitch’ (Type 2, *MYH1* and *MYH2*) skeletal muscle fibres. The dot colour represents gene expression levels in each cell. (c) Proportions of skeletal muscle cell subpopulations in the DM, ASS and NC groups based on scRNA‐seq data. (d) Immunofluorescence staining of IIM muscle tissues with DAPI nuclear staining and antibodies against MYH7, ANKRD2, ISG15, PAX7 and APOC1. MYH7^+^ANKRD2^+^ cells represent Type I fibres, MYH7‐ISG15^+^ cells are ISG^hi^ fibres and PAX7^+^APOC1^+^ cells are MPCs. Circle plots showing ligand–receptor interactions between Type I fibres and T/NK cells (e), Type I fibres and myeloid cell subpopulations (f) and Type I fibres and vascular‐related subtypes (g), as predicted using CellChat analysis. The lines' thickness indicates the ligand–receptor signal's relative strength, with thicker lines representing stronger interactions. The numbers correspond to interaction counts, and the circle sizes are proportional to the number of cells in each group.

We identified four skeletal muscle cell subpopulations, including Type I fibres (*MYH7*, *MYL2* and *ANKRD2*), Type II fibres (*MYH1* and *MYH2*), ISG^hi^ fibres and MPCs (*APOC1*, *MYF5* and *PAX7*) (Figure 4a,b). scRNA‐seq revealed a decrease in Type I fibres and elevated MPCs in both DM and ASS, along with an increase in ISG^hi^ fibres in DM (Figure 4c). Additionally, *ISG15*‐positive fibres comprised 2% in NC, 88.69% in DM and 22.19% in ASS (Figure S8a). *ISG15* mRNA expression and interferon scores were also assessed across the four muscle fibre types, with both measures peaking in the DM group. Consistently, bulk RNA‐seq showed elevated interferon scores in DM and ASS, with no significant change in IMNM (Figure S8b,c). Immunofluorescence confirmed a reduction in Type I fibres (MYH7^+^ANKRD2^+^) in ASS, an increase in ISG^hi^ fibres (MYH7^−^ISG15^+^) in DM and elevated MPCs (PAX7^+^APOC1^+^) in both DM and ASS (Figure 4d).

Given the selective loss of Type I fibres in IIMs (22.66% in ASS vs. 66.68% in NC), we next explored their interactions with inflammatory and vascular‐related cell subsets. Using CellChat, we identified multiple interactions between Type I fibres and these subsets, with the strongest interactions involving CD8 Tcm, CD8 Teff, CD56^dim^CD16^bright^ NK, PPARG^+^ Mφ, SERPINB2^+^ Mo, LYVE1^+^ Mφ, ELPCs and CX3CL1^+^ CECs (Figure 4e–g).

**FIGURE 5:**
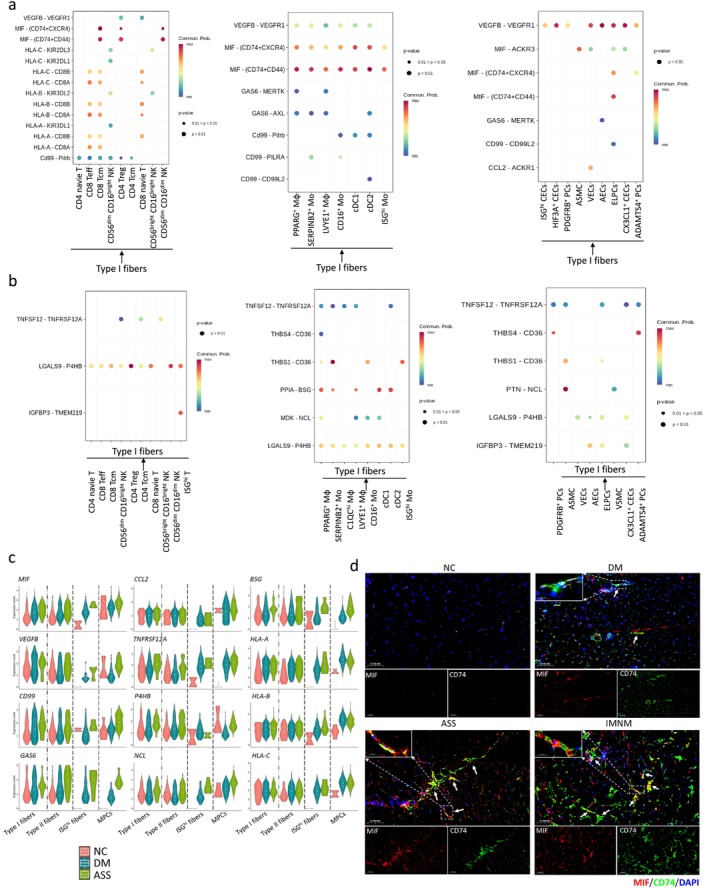
Cell–cell interactions between Type I fibres and immune microenvironment. (a) Bubble plots showing ligand–receptor interactions from Type I fibres to T/NK cells, myeloid cell subpopulations and vascular‐related subtypes. (b) Bubble plots showing ligand–receptor interactions from T/NK cells, myeloid cell subpopulations and vascular‐related subtypes to Type I fibres. Circles are colour‐coded according to the probability of communication, with the size of the circles representing the respective *p* values. (c) Expression of ligand–receptor genes in Type I fibres across IIM subtypes. (d) Immunofluorescence staining showing the localization and expression of MIF (red), CD74 (green) and DAPI (blue, nuclei) in muscle tissues from IIMs.

Next, we analysed specific ligand–receptor pairs involved in these interactions. Type I fibres expressed MIF, along with VEGFB, HLA‐A, HLA‐B, HLA‐C, CD99, GAS6, CCL2, TNFRSF12A, P4HB, NCL and BSG, facilitating communication with inflammatory and vascular‐related cells. In contrast, these inflammatory and vascular cells expressed CD74, CXCR4, LGALS9 and MDK, which in turn interacted with muscle fibres (Figure 5a,b). MIF‐positive fibres accounted for 22.08% in NC, 35.40% in DM and 34.53% in ASS (Figure S9a). We next examined the expression of these ligand/receptor genes across all four fibre types. Most were upregulated in both DM and ASS, with generally higher expression levels in ASS (Figure 5c). Bulk RNA‐seq data from 203 IIM muscle tissue samples further confirmed the upregulation of these ligand–receptor pairs (Figure S9b).

Immunofluorescence staining revealed significantly increased ligand–receptor pair MIF‐CD74 expression in muscle sections from DM, ASS and IMNM patients compared with NC, with exceptionally high levels observed in the necrotic fibres of IMNM patients (Figure 5d).

In summary, we identified selective reductions in Type I fibres, which express specific ligands/receptors that mediate interactions with inflammatory and vascular‐related cells. These findings underscore the complex interplay between muscle fibres and the immune microenvironment in IIMs.

### 3.5 Correlations Between Muscle Tissue Cell Subpopulations and Clinical Indices and IIMs Prognosis

**FIGURE 6:**
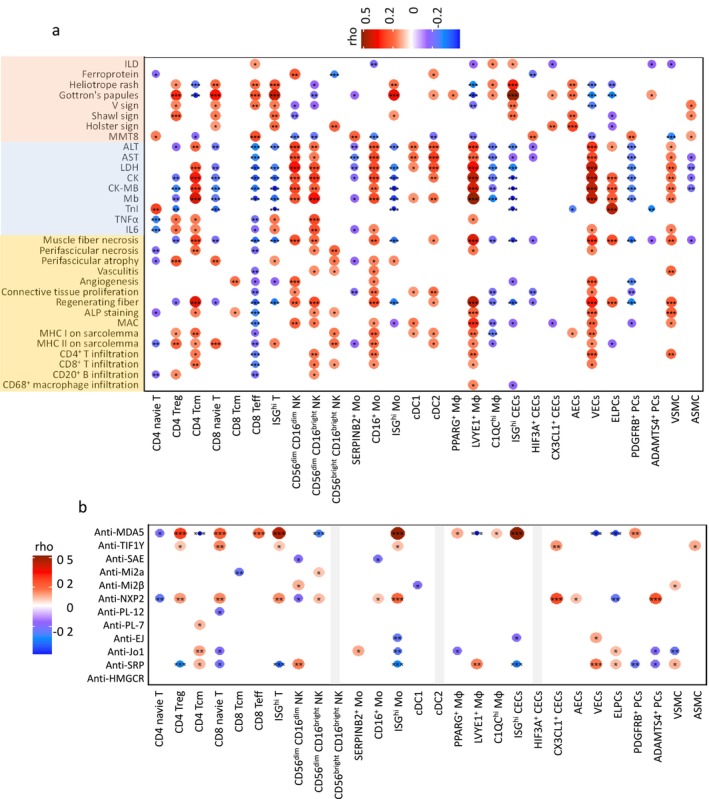
Correlation between the proportion of cell subpopulation, clinical parameters and myositis‐specific antibodies. Correlation between the proportion of cell subtypes and clinical manifestations, laboratory examinations and muscle histopathology. The colours and size of the dots represent the correlation coefficient. *p < 0.05, **p < 0.01, ***p < 0.001. (b) Correlation between the proportion of cell subtypes and autoantibody levels. The colours and size of the dots represent the correlation coefficient, *p < 0.05, **p < 0.01, ***p < 0.001, calculated using Spearman's rank or point‐biserial correlation. Dots represent significant correlations; their absence indicates nonsignificance.

To assess the clinical significance of cell subpopulations in IIM muscle tissues, we performed a correlation analysis between the cell proportions and the clinical manifestations, laboratory findings and histopathological features in the 203 IIMs cohort (Figure 6a). CD8 Teff, C1QC^hi^ Mφ and ISG^hi^ CECs positively correlated with interstitial lung disease (ILD). CD56^dim^CD16^dim^ NK and cDC2 positively correlated with ferroprotein levels. CD4 Treg, CD8 naive T, CD8 Teff, ISG^hi^ T, ISG^hi^ Mo, C1QC^hi^ Mφ, ISG^hi^ CECs and AECs showed positive correlations with various skin rashes (heliotrope rash, Gottron's papules, V sign, shawl sign and holster sign). CD4 Tcm, CD56^dim^CD16^dim^ NK, CD56^dim^CD16^bright^ NK, CD16^+^ Mo, cDC2, LVYE1^+^ Mφ, VECs and VSMC had negative correlations with MMT8 scores but positive correlations with muscle enzymes (alanine transaminase (ALT), aspartate aminotransferase (AST), lactate dehydrogenase (LDH), creatine kinase (CK), myoglobin (Mb)), inflammatory cytokines (tumour necrosis factor [TNF]‐α and IL‐6), myofiber necrosis, regeneration and inflammatory cell infiltration. Conversely, CD8 Teff, SERPINB2^+^ Mo, HIF3A^+^ CECs, PDGFRB^+^ PCs and ASMC showed positive correlations with MMT8 scores but negative correlations with muscle enzymes, inflammatory cytokines, myofiber necrosis, regeneration and inflammatory cell infiltration.

We also explored the relationship between cell subpopulations and MSAs. CD4 Treg, CD8 naive T, ISG^hi^ T and ISG^hi^ Mo were positively correlated with anti‐MDA5, anti‐TIF1γ and anti‐NXP2. Specifically, ISG^hi^ CECs were linked to anti‐MDA5. CD4 Tcm positively correlated with anti‐PL‐7, anti‐Jo1 and anti‐SRP. CD56^dim^CD16^dim^ NK cells were tied to anti‐Mi‐2β and anti‐SRP, whereas CD56^dim^CD16^bright^ NK cells were connected to anti‐Mi‐2α and anti‐NXP2. SERPINB2^+^ Mo was linked to anti‐Jo1. LVYE1^+^ Mφ, VECs, ELPCs and VSMCs also showed associations with anti‐SRP (Figure 6b). To enhance visualization of these correlations, we generated three separate PCA plots illustrating relationships between cell subpopulations and clinical indices, histopathological features and MSAs (Figure S10).

To evaluate the impact of lymphocytes, myeloid cells, vascular‐related cells and key pathways on patient outcomes in IIMs, we applied the Cox proportional hazards model. The follow‐up period was defined as the time from treatment initiation to either the occurrence of a relapse (with death considered a relapse event) or the most recent clinical evaluation. Patient outcomes, including both relapse and nonrelapse cases, were assessed during this period. Relapses were classified as ILD, rash or myositis relapses. ILD relapse was defined by the concurrent presence of worsening respiratory symptoms, radiographic evidence of ILD progression and the initiation or escalation of glucocorticoid or immunosuppressive therapy. Rash and myositis relapses were recorded when recurrent symptoms required intensified treatment [27, 28]. Given the differing treatment responses among IIM subtypes, hazard ratios (HRs) were calculated separately for DM, ASS and IMNM groups.

**FIGURE 7:**
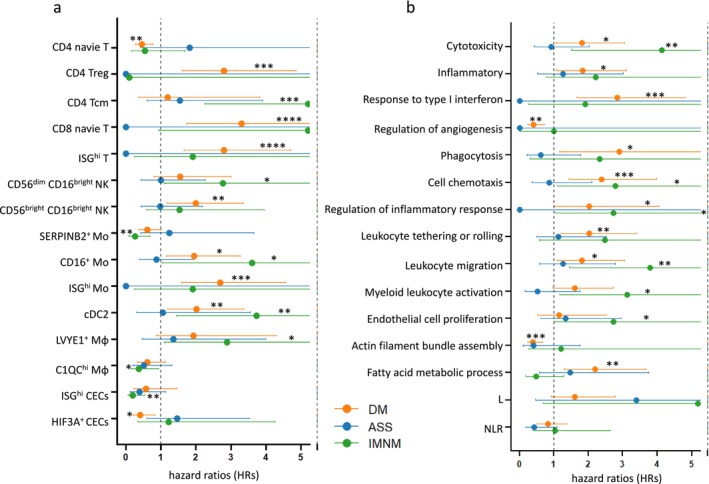
Cellular and molecular predictors of prognosis in IIMs. (a) Forest plot illustrating the associations between specific immune and vascular‐related cell subsets and clinical outcomes in IIM patients. (b) Forest plot highlighting the prognostic significance of pathway activity scores and peripheral blood cell counts. Hazard ratios (HRs) and *p* values were estimated using Cox proportional hazards models. Dots indicate HRs, with error bars representing 95% confidence intervals. *p < 0.05, **p < 0.01, ***p < 0.001, ****p < 0.0001.

Elevated levels of CD4 naive T cells and HIF3A^+^ CECs were associated with favourable outcomes in DM; higher proportions of SERPINB2^+^ Mo, C1QC^hi^ Mφ and ISG^hi^ CECs correlated with better outcomes in IMNM. In contrast, increased levels of CD4 Tregs, CD8 naive T cells, ISG^hi^ T cells, CD56^bright^ CD16^bright^ NK, CD16^+^ Mo, ISG^hi^ Mo and cDC2 were linked to a higher risk of relapse in DM. Similarly, elevated CD4 Tcm, CD56^dim^CD16^bright^ NK, CD16^+^ Mo, cDC2 and LVYE1^+^ Mφ were associated with an increased risk of relapse in IMNM (Figure 7a).

In DM, angiogenesis‐related pathways and actin filament bundle assembly were associated with favourable outcomes. In contrast, higher cytotoxicity scores, inflammatory scores, response to Type I interferon, phagocytosis scores, cell chemotaxis, regulation of inflammatory response, leukocyte tethering and rolling, leukocyte migration and fatty acid metabolic processes were linked to an increased risk of relapse. In IMNM, elevated cytotoxicity scores, cell chemotaxis, regulation of inflammatory response, leukocyte migration, myeloid leukocyte activation and endothelial cell proliferation were associated with a higher relapse risk (Figure 7b).

## 4 Discussion

Our study first defines the cellular architecture of muscle tissues, the immune microenvironment and their interactions across IIM subtypes in the Han Chinese population. It highlights elevated ISG^hi^ T cells, ISG^hi^ Mo and ISG^hi^ CECs in DM. These findings align with previous research linking Type I interferon (IFN‐I) to DM pathogenesis [29, 30, 31]. Although earlier studies on the IFN‐I score primarily focused on anti‐MDA5 DM [29, 30, 31, 32], our analysis reveals that ISG^hi^ cell populations are also present in other DM subgroups, such as anti‐NXP2 and anti‐TIFγ DM. Increased ISG^hi^ T cells and ISG^hi^ Mo in muscle tissues predict higher relapse rates and poorer prognosis in DM. These results suggest that ISG^hi^ cells in muscle tissue may serve as valuable biomarkers for monitoring clinical outcomes in IIMs.

We identified three NK cell subtypes in IIM muscle tissues. Although NK cell development is debated, the CD56^bright^ subset is widely regarded as a precursor to the CD56^dim^ subset. CD56^bright^ NK cells are known for their regulatory and immunomodulatory functions, whereas CD56^dim^ NK cells exhibit more significant cytotoxicity, characterized by higher levels of perforin, granzymes and cytolytic granules, and represent over 90% of NK cells in the blood [33]. Within the CD56^dim^ subset, CD56^dim^CD16^bright^ cells lose CD16 expression upon activation, transitioning into CD56^dim^CD16^dim^/CD16^−^ cells with heightened cytotoxic properties [34]. Our findings indicate that CD56^dim^ NK cells have more cytotoxic potential than CD56^bright^ cells. We observed an increase in CD56^dim^CD16^dim^ NK cells across all IIMs groups, especially in anti‐SRP IMNM, alongside a decrease in CD56^bright^CD16^bright^ cells. We hypothesize that CD56^dim^CD16^dim^ cells represent fully activated NK cells derived from CD56^bright^CD16^bright^ precursors, playing a critical role in muscle damage in IIMs.

We identified various Mo and Mφ in IIM muscle tissues. Decreased levels of SERPINB2^+^ Mo were observed in IIMs. SERPINB2, an inhibitor of the urokinase plasminogen activator, has been linked to monocyte‐derived macrophages (MoMφ). SERPINB2^+^ MoMφ are associated with more prolonged disease‐free survival in patients with colorectal liver metastasis [35]. Our study also showed SERPINB2^+^ Mo was involved in angiogenesis and predicted a better prognosis in IMNM. LYVE1 was associated with resident macrophages in various tissues [36]. LYVE1^+^ Mφ regulates tissue homeostasis by suppressing lung fibrosis and maintaining arterial vessels in the heart. Additionally, perivascular LYVE1^+^ Mφ regulates the angiogenic niche [37]. Elfstrum et al. demonstrated that LYVE1^+^ Mφ represents a tumour‐promoting, anti‐inflammatory subset of macrophages [38]. In our study, LYVE1^+^ Mφ predicted a worse prognosis in IMNM.

Vascular‐related cells exhibit distinct patterns across IIM subtypes. We identified a novel cluster of HIF3A^+^ CECs in muscle tissues. HIF3A regulates the adaptive response to low oxygen and hypoxia‐inducible gene expression. In pulmonary endothelial cells, HIF3A promotes angiogenesis by repressing VE‐cadherin gene expression [39]. The reduced HIF3A^+^ CECs in IIMs may be linked to impaired angiogenesis in muscle damage. Additionally, a high proportion of HIF3A^+^ CECs predicts a better prognosis in DM. We also found increased ELPCs in ASS and IMNM groups. ELPCs expressing pericyte and pan‐endothelial genes may represent a transition between endothelial cells and pericytes [40]. ELPCs positively correlate with muscle enzymes and perifascicular necrosis, indicating a role in muscle tissue repair.

We found that Type I fibres were selectively reduced in IIMs and exhibited high expression of MIF, MHC I molecules and multiple ligand–receptor pairs, particularly in DM and ASS, suggesting active interaction with the immune microenvironment. MIF is a pleiotropic cytokine with pro‐inflammatory, pro‐proliferative and chemotactic properties, playing key roles in tissue migration and in the recruitment and retention of inflammatory cells [41]. The expression of MIF and its receptor CD74 was significantly increased in the muscle tissues of IIM patients. These findings suggest that MIF may serve as a promising therapeutic target for IIMs. However, the precise mechanisms by which MIF contributes to disease pathogenesis remain poorly understood.

One limitation of our study is that some NC samples were derived from the quadriceps femoris, whereas all IIM samples were obtained from the biceps brachii. Although anatomical differences between these muscles may influence muscle fibre subtypes, we did not perform deconvolution specifically on muscle fibres. Nonetheless, CIBERSORTx analysis of NC samples from both muscle groups revealed no significant differences in immune or vascular‐related cell subpopulations.

Additionally, although our scRNA‐seq sample size is limited, we captured sufficient numbers of immune cells, vascular‐related cells and muscle fibres, allowing for robust classification into distinct subtypes using established marker genes. However, we did not base our analysis on the proportions derived from scRNA‐seq data for the following reasons: (1) The capture rate of individual cells during mechanical dissociation or enzymatic digestion varies across cell types and subpopulations. (2) scRNA‐seq introduces biases in single‐cell recovery due to variations in cell sorting parameters, such as sheath fluid flow rate, core stream flow rate and nozzle size. For example, 10X Genomics' Chromium scRNA‐seq typically recovers only 50%–65% of loaded cells, with over 35% lost during the loading process, affecting the accuracy of cell‐composition analyses. (3) Filtering criteria for removing doublets or low‐quality samples significantly influence results, with varying thresholds leading to divergent outcomes. To minimise potential biases, we used CIBERSORTx, a robust deconvolution tool commonly referred to as ‘digital cytometry’, to estimate cell proportions in a larger cohort of 203 IIM patients. To validate the reliability of these estimates, we compared the results with those obtained from scRNA‐seq, GSEA and immunofluorescence. These complementary approaches demonstrated strong concordance and further supported the accuracy of the CIBERSORTx analysis. Looking ahead, advanced techniques such as multiplexed error‐robust fluorescence in situ hybridization may enable direct functional investigation of cell subpopulations within IIM muscle tissues.

## Ethics Statement

This study involves human participants and was approved by the ethical committee of the Xiangya Hospital, Central South University. ID: 201303293. Before participating, participants gave informed consent.

## Consent

The authors have nothing to report.

## Conflicts of Interest

The authors declare no conflicts of interest.

## Supporting information


**Figure S1.** Single‐cell atlas of cell types in idiopathic inflammatory myopathies (IIMs) muscle tissues. (a) A total of 34 972 high‐quality cells from three normal control (NC), three dermatomyositis (DM) and three antisynthetase syndrome (ASS) muscle tissues are projected using a uniform manifold approximation and projection (UMAP) plot. Colours indicate contour curves that outline the major cell types and cluster boundaries. The axis outside the circular plot depicts the log scale of the total cell number for each cell class. The three coloured tracks (from outside to inside) indicate the cluster (coloured as in the central UMAP), group and sample. The legend shows the group/colours for the group track. (b) Dot plot showing the marker genes characterizing each main cluster, with selected genes listed. Dot size corresponds to the percentage of cells in a cluster expressing the gene, and dot colour corresponds to the average gene expression level in the cluster.
**Figure S2.** Characterization and functional profiling of T and NK cell subpopulations in IIMs. (a) Dot plot showing the marker genes characterizing each T and NK cell subpopulation, with selected genes listed. Dot size corresponds to the percentage of cells in a cluster expressing the gene, and dot colour corresponds to the average gene expression level in the cluster. (b) The percentage of CD4^+^ and CD8^+^ ISG^hi^ T cells in the scRNA‐seq data from six IIMs and three NC. (c) Gene Ontology (GO) enrichment analysis was performed on the top marker genes in ISG^hi^ T cells. (d) Violin plots illustrate cytotoxicity, inflammation and interferon scores in lymphocyte subpopulations across IIM subtypes. (e) Percentage of lymphocyte subpopulations in the DM, ASS and NC groups from scRNA‐seq data. (f) Gene set enrichment analysis (GSEA) plot of the lymphocyte subpopulations gene sets in the IIMs compared to HC. The colours and size of the dots represent the normalized enrichment score (NES). *p < 0.05, **p < 0.01, ***p < 0.001.
**Figure S3.** Distribution of lymphocyte subpopulations in muscle tissues across IIMs antibody subgroups by CIBERSORTx. (a) Immunofluorescence identified CD4^+^, CD8^+^ and double‐negative (CD4^−^CD8^−^) ISG^hi^ T cells in DM muscle tissues. (b) Relative proportions of individual lymphocyte subpopulations in the muscle tissues of IIMs antibody subpopulations and NC as determined by CIBERSORTx analysis. *p < 0.05, **p < 0.01, ***p < 0.001, ****p < 0.0001 by unpaired *t*‐test (for normally distributed data) or Wilcoxon test (for nonnormally distributed data).
**Figure S4.** Characterization of myeloid cell subpopulations in IIMs. (a) Dot plot illustrating marker genes that characterize each myeloid cell subpopulation, with selected genes listed. Dot size indicates the percentage of cells in a cluster expressing the gene, and dot colour represents the average gene expression level in the cluster. (b) Violin plots depicting the activity of key pathways in myeloid cell subpopulations across different IIM subtypes.
**Figure S5.** Myeloid cell subpopulation analysis in muscle tissues of IIMs antibody subgroups using CIBERSORTx. (a) Percentage of myeloid cell subpopulations among DM, ASS and NC groups from scRNA‐seq data. (b) GSEA plot depicting enrichment of gene sets specific to myeloid cell subpopulations in IIMs compared to HC. The colours and size of the dots represent the NES. *p < 0.05, **p < 0.01, ***p < 0.001. (c) A comparison of relative proportions of individual myeloid cell subpopulations in muscle tissues among IIMs antibody subgroups and NC was analysed using CIBERSORTx. Statistical significance was indicated by *p < 0.05, **p < 0.01, ***p < 0.001, ****p < 0.0001, determined by unpaired *t*‐test (for normally distributed data) or Wilcoxon test (for nonnormally distributed data).
**Figure S6.** Molecular and functional profiling of vascular‐related cell subpopulations in IIMs. (a) Dot plot depicting marker genes characterizing each vascular‐related cell subpopulation, with selected genes listed. Dot size indicates the percentage of cells in a cluster expressing the gene, and dot colour corresponds to the average gene expression level in the cluster. (b) Violin plots illustrating the activity of key pathways in vascular‐related cell subpopulations across different IIM subtypes.
**Figure S7.** Vascular‐related cell subpopulation profiling in muscle tissues of IIMs antibody subgroups by CIBERSORTx. (a) Distribution of vascular‐related cell subpopulations among DM, ASS and NC groups from scRNA‐seq data. (b) GSEA plot depicting enrichment of gene sets specific to vascular‐related cell subpopulations in IIMs compared to NC. The colours and size of the dots represent the NES. *p < 0.05, **p < 0.01, ***p < 0.001. (c) Comparative analysis of the relative proportions of individual vascular‐related cell subpopulations in muscle tissues among IIMs antibody subgroups and NC, analysed using CIBERSORTx. Significance levels were indicated by *p < 0.05, **p < 0.01, ***p < 0.001, ****p < 0.0001, determined by unpaired *t*‐test (for normally distributed data) or Wilcoxon test (for nonnormally distributed data).
**Figure S8.** Distribution of ISG15‐positive muscle fibres and interferon scores across IIM subtypes. (a) Proportion of ISG15‐positive muscle fibres in DM, ASS and NC groups based on scRNA‐seq data. (b) Violin plots showing ISG15 expression and interferon scores in muscle fibres across IIM subtypes. (c) Box plot illustrating interferon scores derived from bulk RNA‐seq data across different IIM subtypes. **p < 0.01, ****p < 0.0001, determined by the Wilcoxon test.
**Figure S9.** MIF‐positive muscle fibres and ligand–receptor gene expression across IIM subtypes. (a) Proportion of MIF‐positive muscle fibres in DM, ASS and NC groups based on scRNA‐seq data. (b) Expression levels of ligand and receptor genes in muscle tissues from IIM patients. *p < 0.05, **p < 0.01, ***p < 0.001, ****p < 0.0001, determined by unpaired *t*‐test (for normally distributed data) or Wilcoxon test (for nonnormally distributed data). TPM: transcripts per kilobase million.
**Figure S10.** Correlations between cell subpopulations and clinical indices, histopathological features and MSAs. PCA plots illustrating the relationships between immune and vascular‐related cell subpopulations and (a) clinical indices, (b) histopathological features and (c) MSAs. Immune and vascular‐related cell subpopulations are shown in red, whereas clinical indices, histopathological features and MSAs are shown in blue.
**Table S1.** Comparison of clinical manifestations and laboratory data among IIMs and NC for Bulk RNA sequencing.
**Data S1.** List of marker genes used for GSEA.

## Data Availability

Data are available upon reasonable request.

## References

[1] I. E. Lundberg , M. Fujimoto , J. Vencovsky , et al., “Idiopathic Inflammatory Myopathies,” Nature Reviews Disease Primers 7 (2021): 86. 10.1038/s41572-021-00321-x 34857798

[2] C. Franco , M. Gatto , L. Iaccarino , A. Ghirardello , and A. Doria , “Lymphocyte Immunophenotyping in Inflammatory Myositis: A Review,” Current Opinion in Rheumatology 33 (2021): 522–528. 34402455 10.1097/BOR.0000000000000831

[3] A. Uruha , H. H. Goebel , and W. Stenzel , “Updates on the Immunopathology in Idiopathic Inflammatory Myopathies,” Current Rheumatology Reports 23, no. 7 (2021): 56. 34212266 10.1007/s11926-021-01017-7

[4] C. Preusse , B. Paesler , C. Nelke , et al., “Skeletal Muscle Provides the Immunological Micro‐Milieu for Specific Plasma Cells in Anti‐Synthetase Syndrome‐Associated Myositis,” Acta Neuropathologica 144 (2022): 353–372. 35612662 10.1007/s00401-022-02438-z PMC9288384

[5] A. Argyriou , B. Horuluoglu , A. S. Galindo‐Feria , et al., “Single‐Cell Profiling of Muscle‐Infiltrating T Cells in Idiopathic Inflammatory Myopathies,” EMBO Molecular Medicine 15 (2023): e17240. 37522383 10.15252/emmm.202217240 PMC10565639

[6] R. Lahoria , D. Selcen , and A. G. Engel , “Microvascular Alterations and the Role of Complement in Dermatomyositis,” Brain 139 (2016): 1891–1903. 27190020 10.1093/brain/aww122

[7] D. Lemmer , J. Schmidt , K. Kummer , et al., “Impairment of Muscular Endothelial Cell Regeneration in Dermatomyositis,” Frontiers in Neurology 13 (2022): 952699. 36330424 10.3389/fneur.2022.952699 PMC9623165

[8] M. Honda , F. Shimizu , R. Sato , et al., “Jo‐1 Antibodies From Myositis Induce Complement‐Dependent Cytotoxicity and TREM‐1 Upregulation in Muscle Endothelial Cells,” Neurology Neuroimmunology & Neuroinflammation 10 (2023): e200116. 37147138 10.1212/NXI.0000000000200116 PMC10162704

[9] J. Wienke , L. M. Pachman , G. A. Morgan , et al., “Endothelial and Inflammation Biomarker Profiles at Diagnosis Reflecting Clinical Heterogeneity and Serving as a Prognostic Tool for Treatment Response in Two Independent Cohorts of Patients With Juvenile Dermatomyositis,” Arthritis & Rhematology 72 (2020): 1214–1226. 10.1002/art.41236 PMC7329617 32103637

[10] A. L. Mammen , Y. Allenbach , W. Stenzel , O. Benveniste , and ENMC 239th Workshop Study Group . 2020. “239th ENMC International Workshop: Classification of Dermatomyositis, Amsterdam, the Netherlands, 14–16 December 2018.” Neuromuscular Disorders 30, no. 1: 70–92. 31791867 10.1016/j.nmd.2019.10.005

[11] K. Li , C. Pu , X. Huang , J. Liu , Y. Mao , and X. Lu . 2015. “Clinicopathologic Features of Sporadic Inclusion Body Myositis in China.” Neurologia i Neurochirurgia Polska 49, no. 4: 245–250. 26188941 10.1016/j.pjnns.2015.06.004

[12] A. L. Mammen , Y. Allenbach , W. Stenzel , O. Benveniste , and Group EtWS , “239th ENMC International Workshop: Classification of Dermatomyositis, Amsterdam, the Netherlands, 14–16 December 2018,” Neuromuscular Disorders 30 (2020): 70–92. 31791867 10.1016/j.nmd.2019.10.005

[13] G. R. Connors , L. Christopher‐Stine , C. V. Oddis , and S. K. Danoff , “Interstitial Lung Disease Associated With the Idiopathic Inflammatory Myopathies: What Progress Has Been Made in the Past 35 Years?,” Chest 138 (2010): 1464–1474. 21138882 10.1378/chest.10-0180

[14] W. Stenzel , A. L. Mammen , L. Gallay , et al., “273rd ENMC International Workshop: Clinico‐Sero‐Morphological Classification of the Antisynthetase Syndrome. Amsterdam, the Netherlands, 27–29 October 2023,” Neuromuscular Disorders 45 (2024): 104453. 39490006 10.1016/j.nmd.2024.104453

[15] Y. Allenbach , A. L. Mammen , O. Benveniste , W. Stenzel , and Immune‐Mediated Necrotizing Myopathies Working G , “224th ENMC International Workshop: Clinico‐Sero‐Pathological Classification of Immune‐Mediated Necrotizing Myopathies Zandvoort, the Netherlands, 14–16 October 2016,” Neuromuscular Disorders 28, no. 1 (2018): 87–99. 29221629 10.1016/j.nmd.2017.09.016

[16] G. Chen , B. Ning , and T. Shi , “Single‐Cell RNA‐Seq Technologies and Related Computational Data Analysis,” Frontiers in Genetics 10 (2019): 317. 31024627 10.3389/fgene.2019.00317 PMC6460256

[17] B. Hwang , J. H. Lee , and D. Bang , “Single‐Cell RNA Sequencing Technologies and Bioinformatics Pipelines,” Experimental and Molecular Medicine 50 (2018): 1–14. 10.1038/s12276-018-0071-8 PMC6082860 30089861

[18] B. Chen , M. S. Khodadoust , C. L. Liu , A. M. Newman , and A. A. Alizadeh , “Profiling Tumor Infiltrating Immune Cells With CIBERSORT,” Methods in Molecular Biology 1711 (2018): 243–259. 29344893 10.1007/978-1-4939-7493-1_12 PMC5895181

[19] R. L. Belote , D. Le , A. Maynard , et al., “Human Melanocyte Development and Melanoma Dedifferentiation at Single‐Cell Resolution,” Nature Cell Biology 23 (2021): 1035–1047. 34475532 10.1038/s41556-021-00740-8

[20] C. Cascini and C. Chiodoni , “The Immune Landscape of Osteosarcoma: Implications for Prognosis and Treatment Response,” Cells 10, no. 7 (2021): 1668. 34359840 10.3390/cells10071668 PMC8304628

[21] L. Wang , F. Chen , R. Liu , L. Shi , G. Zhao , and Z. Yan , “Gene Expression and Immune Infiltration in Melanoma Patients With Different Mutation Burden,” BMC Cancer 21 (2021): 379. 33836680 10.1186/s12885-021-08083-1 PMC8034108

[22] A. M. Newman , C. L. Liu , M. R. Green , et al., “Robust Enumeration of Cell Subsets From Tissue Expression Profiles,” Nature Methods 12 (2015): 453–457. 25822800 10.1038/nmeth.3337 PMC4739640

[23] A. M. Newman , C. B. Steen , C. L. Liu , et al., “Determining Cell Type Abundance and Expression From Bulk Tissues With Digital Cytometry,” Nature Biotechnology 37, no. 7 (2019): 773–782. 10.1038/s41587-019-0114-2 PMC6610714 31061481

[24] C. B. Steen , C. L. Liu , A. A. Alizadeh , and A. M. Newman , “Profiling Cell Type Abundance and Expression in Bulk Tissues With CIBERSORTx,” Methods in Molecular Biology 2117 (2020): 135–157. 31960376 10.1007/978-1-0716-0301-7_7 PMC7695353

[25] H. Zhu , H. Luo , B. Skaug , et al., “Fibroblast Subpopulations in Systemic Sclerosis: Functional Implications of Individual Subpopulations and Correlations With Clinical Features,” Journal of Investigative Dermatology 144 (2024): 1251–61e13. 38147960 10.1016/j.jid.2023.09.288 PMC11116078

[26] D. Y. Vogel , J. E. Glim , A. W. Stavenuiter , et al., “Human Macrophage Polarization In Vitro: Maturation and Activation Methods Compared,” Immunobiology 219 (2014): 695–703. 24916404 10.1016/j.imbio.2014.05.002

[27] X. Chen , L. Zhang , Q. Jin , et al., “The Clinical Features and Prognoses of Anti‐MDA5 and Anti‐Aminoacyl‐tRNA Synthetase Antibody Double‐Positive Dermatomyositis Patients,” Frontiers in Immunology 13 (2022): 987841. 36110863 10.3389/fimmu.2022.987841 PMC9468482

[28] H. Yang , L. Zhang , X. Tian , et al., “Distinct Phenotype and Prognosis of Immune‐Mediated Necrotizing Myopathy Based on Clinical‐Serological‐Pathological Classification,” Rheumatology 64 (2024): 2252–2264. 10.1093/rheumatology/keae361 39029922

[29] Y. Ye , Z. Chen , S. Jiang , et al., “Single‐Cell Profiling Reveals Distinct Adaptive Immune Hallmarks in MDA5+ Dermatomyositis With Therapeutic Implications,” Nature Communications 13 (2022): 6458. 10.1038/s41467-022-34145-4 PMC9617246 36309526

[30] X. Chen , D. Lian , and H. Zeng , “Single‐Cell Profiling of Peripheral Blood and Muscle Cells Reveals Inflammatory Features of Juvenile Dermatomyositis,” Frontiers in Cell and Developmental Biology 11 (2023): 1166017. 37152289 10.3389/fcell.2023.1166017 PMC10157079

[31] S. A. Greenberg , J. L. Pinkus , G. S. Pinkus , et al., “Interferon‐Alpha/Beta‐Mediated Innate Immune Mechanisms in Dermatomyositis,” Annals of Neurology 57 (2005): 664–678. 15852401 10.1002/ana.20464

[32] S. A. Greenberg , B. W. Higgs , C. Morehouse , et al., “Relationship Between Disease Activity and Type 1 Interferon‐ and Other Cytokine‐Inducible Gene Expression in Blood in Dermatomyositis and Polymyositis,” Genes and Immunity 13 (2012): 207–213. 21881594 10.1038/gene.2011.61

[33] S. Weber , K. B. Menees , J. Park , et al., “Distinctive CD56(Dim) NK Subset Profiles and Increased NKG2D Expression in Blood NK Cells of Parkinson's Disease Patients,” NPJ Parkinson's Disease 10 (2024): 36. 10.1038/s41531-024-00652-y PMC10869354 38360903

[34] R. Romee , B. Foley , T. Lenvik , et al., “NK Cell CD16 Surface Expression and Function Is Regulated by a Disintegrin and Metalloprotease‐17 (ADAM17),” Blood 121 (2013): 3599–3608. 23487023 10.1182/blood-2012-04-425397 PMC3643761

[35] N. Cortese , R. Carriero , M. Barbagallo , et al., “High‐Resolution Analysis of Mononuclear Phagocytes Reveals GPNMB as a Prognostic Marker in Human Colorectal Liver Metastasis,” Cancer Immunology Research 11 (2023): 405–420. 36652202 10.1158/2326-6066.CIR-22-0462 PMC10070171

[36] N. W. Gale , R. Prevo , J. Espinosa , et al., “Normal Lymphatic Development and Function in Mice Deficient for the Lymphatic Hyaluronan Receptor LYVE‐1,” Molecular and Cellular Biology 27 (2007): 595–604. 17101772 10.1128/MCB.01503-06 PMC1800809

[37] J. W. Opzoomer , J. E. Anstee , I. Dean , et al., “Macrophages Orchestrate the Expansion of a Proangiogenic Perivascular Niche During Cancer Progression,” Science Advances 7 (2021): eabg9518. 34730997 10.1126/sciadv.abg9518 PMC8565907

[38] A. K. Elfstrum , A. H. Rumahorbo , L. E. Reese , E. V. Nelson , B. M. McCluskey , and K. L. Schwertfeger , “LYVE‐1‐Expressing Macrophages Modulate the Hyaluronan‐Containing Extracellular Matrix in the Mammary Stroma and Contribute to Mammary Tumor Growth,” Cancer Research Communications 4 (2024): 1380–1397. 38717149 10.1158/2767-9764.CRC-24-0205 PMC11141485

[39] S. Kobayashi , T. Yamashita , K. Ohneda , et al., “Hypoxia‐Inducible Factor‐3α Promotes Angiogenic Activity of Pulmonary Endothelial Cells by Repressing the Expression of the VE‐Cadherin Gene,” Genes to Cells 20 (2015): 224–241. 25626335 10.1111/gtc.12215

[40] A. Cameron , G. Wakelin , N. Gaulton , et al., “Identification of Underexplored Mesenchymal and Vascular‐Related Cell Populations in Human Skeletal Muscle,” American Journal of Physiology. Cell Physiology 323 (2022): C1586–C1600. 36342160 10.1152/ajpcell.00364.2022

[41] A. Hoffmann , L. C. Zwissler , O. El Bounkari , and J. Bernhagen , “Studying the Pro‐Migratory Effects of MIF,” Methods in Molecular Biology 2080 (2020): 1–18. 31745866 10.1007/978-1-4939-9936-1_1

