# Supplementary material for: Characterization of Muscle Tissue Cell Diversity and Clinical Implications in Idiopathic Inflammatory Myopathy
Source: J Cachexia Sarcopenia Muscle. 2025 Aug 28;16(5):e70043. doi: 10.1002/jcsm.70043 (PMC12391830; doi:10.1002/jcsm.70043)
Supplement: Supplementary file 1 — Figure S1. Single‐cell atlas of cell types in idiopathic inflammatory myopathies (IIMs) muscle tissues. (a) A total of 34 972 high‐quality cells from three normal control (NC), three dermatomyositis (DM) and three antisynthetase syndrome (ASS) muscle tissues are projected using a uniform manifold approximation and projection (UMAP) plot. Colours indicate contour curves that outline the major cell types and cluster boundaries. The axis outside the circular plot depicts the log scale of the total cell number for each cell class. The three coloured tracks (from outside to inside) indicate the cluster (coloured as in the central UMAP), group and sample. The legend shows the group/colours for the group track. (b) Dot plot showing the marker genes characterizing each main cluster, with selected genes listed. Dot size corresponds to the percentage of cells in a cluster expressing the gene, and dot colour corresponds to the average gene expression level in the cluster. Figure S2. Characterization and functional profiling of T and NK cell subpopulations in IIMs. (a) Dot plot showing the marker genes characterizing each T and NK cell subpopulation, with selected genes listed. Dot size corresponds to the percentage of cells in a cluster expressing the gene, and dot colour corresponds to the average gene expression level in the cluster. (b) The percentage of CD4+ and CD8+ ISGhi T cells in the scRNA‐seq data from six IIMs and three NC. (c) Gene Ontology (GO) enrichment analysis was performed on the top marker genes in ISGhi T cells. (d) Violin plots illustrate cytotoxicity, inflammation and interferon scores in lymphocyte subpopulations across IIM subtypes. (e) Percentage of lymphocyte subpopulations in the DM, ASS and NC groups from scRNA‐seq data. (f) Gene set enrichment analysis (GSEA) plot of the lymphocyte subpopulations gene sets in the IIMs compared to HC. The colours and size of the dots represent the normalized enrichment score (NES). *p < 0.05, **p < 0.01, ***p < 0.001 [file JCSM-16-e70043-s001.pdf]

Supplementary Material

Characterization of muscle tissue cell diversity and clinical implications in idiopathic inflammatory myopathy

Honglin Zhu*[1,2,3], Yizhi Xiao[1,2,3], Shasha Xie[1,2,3], Qiming Meng[1,2,3], Ting Ding[1,2,3], Ting Huang[1,2,3], Di Liu[1,2,3], Sijia Liu[1,2,3], Xiaoli Zhang[1,2,3], Huali Zhang[1,4], Hui Luo*[1,2,3]

1 Department of Rheumatology and Immunology, Xiangya Hospital, Central South University, Changsha, Hunan, P.R. China

2 National Clinical Research Center for Geriatric Disorders, Xiangya Hospital, Central South University, Changsha, Hunan, P.R. China

3 Provincial Clinical Research Center for Rheumatic and Immunologic Diseases, Xiangya Hospital, Changsha, Hunan, P.R. China

4 Department of Pathophysiology, Xiangya School of Medicine, Central South University, Changsha, Hunan, P.R. China

*** Corresponding authors:**

Honglin Zhu; Xiangya Hospital, Central South University, 87 Xiangya Road, Changsha, Hunan 410008, China. Tel: +86 731 89753548. Fax: +86 731 89753548.

E-mail: Honglinzhu@csu.edu.cn;

Hui Luo; Xiangya Hospital, Central South University, 87 Xiangya Road, Changsha, Hunan 410008, China. Tel: +86 731 89753548. Fax: +86 731 89753548.

E-mail: luohui@csu.edu.cn

Supplementary Materials and Methods

Single-cell RNA sequencing (scRNA-seq) sample preparation and data analysis

(1) Sample processing

Muscle tissues for scRNA-seq were obtained from 3 dermatomyositis (DM), 3 anti-synthetase syndrome (ASS), and 3 normal control (NC). The collected tissues were digested enzymatically with collagenase II for 2 hours and further dispersed using Ham's F-10 Nutrient Mixture (Gibco) supplemented with 5% horse serum. The resulting cell suspension was filtered twice through 70 μm cell strainers and resuspended in PBS containing 0.04% BSA. Qualified cell suspensions were then used for further scRNA-seq analysis[1].

(2) Single-cell library preparation and RNA sequencing

Qualified cell suspensions from muscle biopsies were loaded into chromium microfluidic chips with 3' v3 chemistry and barcoded with a 10× Chromium Controller (10x Genomics GemCode Technology, CA). RNA from the barcoded cells was subsequently reverse-transcribed, and sequencing libraries were constructed with a Chromium Single Cell 3' v3 reagent kit according to the manufacturer's instructions. Sequencing was performed with the Illumina NovaSeq 6000 platform according to the manufacturer's instructions (Illumina, Inc., San Diego, CA, USA).

(3) scRNA-seq data analysis

The raw scRNA-seq data were processed using Cell Ranger 7.0.0 (10x Genomics). The reads were mapped to the human genome GRCh38 to generate feature-barcode matrices. Data analysis was performed with the R package Seurat V4.0.0. Cells were first filtered by the number of detected UMIs per cell (nUMI; ≥ 600), the number of detected genes (nGene; ≥ 300), log10GenesPerUMI (> 0.80), and mitochondrial genes observed/total number of genes observed (mitoRatio; < 0.2). Sctransform was used for data normalization, and reciprocal principal component analysis (RPCA) were used to integrate data from all samples [2]. Principal component analysis (PCA) and uniform manifold approximation and projection (UMAP) were used for visualization. The main clusters were first annotated by SingleR (v1.4.0) with reference datasets

from HumanPrimaryCellAtlasData and BlueprintEncodeData from the Celldex package (v1.0.0)[3] and confirmed by classical marker genes, such as *CD3D*, *CD19*, *CD68*, *CD14*, *LYZ*, *VWF*, *COL1A1*, *ACTA2*, *CDH15*, *MYF5*, and *PAX7*, for immune cells, endothelial cells, fibroblasts and skeletal muscle cells. To achieve a more precise definition of individual cell subtypes, lymphocytes, myeloid cells, vascular-related cells, and skeletal muscle cells were reclustered separately.

Gene expression was visualized using UMAP, dot plots, violin plots, and heatmaps, which were generated with the Seurat functions DimPlot, FeaturePlot, DotPlot, VlnPlot, and DoHeatmap. Signature marker genes for each cluster were identified using the FindAllMarkers function in Seurat with the MAST test. The activity levels of cytotoxicity, inflammation, M1 macrophages, M2 macrophages, angiogenesis, stress genes and phagocytosis for each cell were scored using AUCell with established marker genes[4, 5]. The genes used to calculate the interferon response score were sourced from the Molecular Signatures Database, specifically from 'REACTOME_INTERFERON_SIGNALING'[6]. Gene Ontology (GO) enrichment analysis was performed for the top 200 enriched marker genes using the enrichGO function from the R package clusterProfiler. Using the Benjamini- Hochberg procedure, *p* values were computed with a hypergeometric test and adjusted for multiple hypothesis testing. Bubble plots displaying the enriched GO terms were created with ggplot2.

Bulk RNA-seq of muscle tissues from idiopathic inflammatory myopathies (IIMs) and NC

Muscle biopsies (203 IIMs, 19 NC) were immediately frozen and stored in liquid nitrogen. Then, total RNA from muscle tissues was extracted using TRIzol reagent (Invitrogen Life Technologies, California, USA) according to the manufacturer's protocol. RNA-seq was performed by Novogene (Beijing, China) on an Illumina NovaSeq platform using a paired-end 150 bp sequencing strategy. FastQC software (v0.11.8) and Trimmomatic tools (v0.38) were used to check the sequencing reads

quality and to obtain clean reads. Samtools (v1.9) and hisat2 (v2.1.0) were used for alignment and mapping of sequencing reads to the human reference genome hg38. Gene annotation was performed by subread (v1.6.3) with gencode.v35.annotation.gtf. The final count data were used for CIBERSORTx analysis[7].

Calculating the abundance of individual cell subtypes from bulk transcriptome datasets using CIBERSORTx

The abundance of individual lymphocyte, myeloid, and vascular-related cell subpopulations was determined using CIBERSORTx, a well-established machine-learning method for estimating the proportions of specific cell types from bulk transcriptomes based on transcriptional profiles obtained through scRNA-seq. CIBERSORTx has been widely applied across various cell types and tissues. Initially, we generated a single gene signature matrix using the "Create Signature Matrix" module in CIBERSORTx, based on our own scRNA-seq dataset, which included cells from both IIMs and control samples. This unified matrix was applied to all bulk RNA-seq samples—regardless of disease status—using high-resolution mode. This approach follows the recommended framework outlined in the original CIBERSORTx publication[7], which supports the use of a single matrix derived from biologically diverse conditions for cell type deconvolution. We then applied mode batch correction and performed 1,000 permutations with quantile normalization disabled to analyze the relative cell type fractions and calculate the proportions of each cell subtype from the IIMs bulk transcriptomes[7, 8].

Gene Set Enrichment Analysis (GSEA)

To validate and confirm the results from CIBERSORTx, we employed an alternative approach using Gene Set Enrichment Analysis (GSEA) with the R package clusterProfiler (v3.18.0). We utilized the top marker genes as signature genes to derive the enrichment scores. The marker gene lists are provided in Supplementary Data 1.

Inference of Cell-Cell Interactions

The interactions between Type I fibers and T/NK cells, myeloid cell subpopulations, and vascular-related subtypes were analyzed using scRNA-seq data and the CellChat (V2) algorithm[9]. The communication probabilities and biologically significant cell-cell communication numbers were computed, and the ligand-receptor pairs were visualized.

Immunofluorescence staining

Immunofluorescence staining of the muscle tissues was performed on frozen sections. All slides were fixed with 4% paraformaldehyde. After three times washes, the slides were permeabilized with 0.1% Triton X-100 in PBS for 15 min, blocked with 5% goat serum in PBS for 30 min, and then incubated overnight at 4°C with mouse anti-human antibodies against Dystrophin (1:50, Santa Cruz, USA), PAX7(1:100, Santa Cruz, USA), APOC1, MYH7, ANKRD2, ISG15, MIF (all used 1:100, Abcam, USA), CD68 (1:400, Abcam, USA), CD3 (1:1000, Abcam, USA), CD4, CD8, CD74 (all used 1:500, Cell Signaling Technology, USA), C1QC (1:400, Proteintech, USA), FITC anti-human CD3 (1:50, BioLegend, USA). The corresponding secondary antibodies were incubated for 1 h at room temperature in the dark, and antifading mounting medium with 4’,6-diamidino-2-phenylindole (DAPI, Solarbio, Beijing, China) was then added. Unrelated isotype-matched antibodies were used as controls. Images were captured using an Olympus microscope with a DP72 camera (Olympus, Shinjuku, Japan). Each staining experiment was repeated three times with samples from different IIMs patients and NC, and representative stained sections were shown.

Survival analysis

The follow-up period was defined as the time from treatment initiation to either the occurrence of a relapse (with death considered a relapse event) or the most recent clinical evaluation. Among the 203 patients in our IIMs cohort, complete follow-up data were available for 187, while 16 were lost to follow-up. The follow-up duration

ranged from 0.5 to 89 months. Patient outcomes—including both relapse and non-relapse cases—were assessed during this period. Relapses were categorized as ILD, rash, or myositis relapses. ILD relapse was defined by the concurrent presence of worsening respiratory symptoms, radiographic evidence of ILD progression, and the initiation or escalation of glucocorticoids or immunosuppressive therapy. Rash and myositis relapses were recorded when recurrent symptoms necessitated intensified treatment[10, 11]. We used the Cox proportional hazards model from the survival package to examine the correlation between lymphocytes, myeloid cells, vascular-related cells, and patient outcomes, and calculated the hazard ratios (HRs).

Reference

1. Charville GW, Cheung TH, Yoo B, Santos PJ, Lee GK, Shrager JB, et al. Ex Vivo Expansion and In Vivo Self-Renewal of Human Muscle Stem Cells. Stem Cell Reports. 2015;5:621-32.
2. Stuart T, Butler A, Hoffman P, Hafemeister C, Papalexi E, Mauck WM, 3rd, et al. Comprehensive Integration of Single-Cell Data. Cell. 2019;177:1888-902 e21.
3. Aran D, Looney AP, Liu L, Wu E, Fong V, Hsu A, et al. Reference-based analysis of lung single-cell sequencing reveals a transitional profibrotic macrophage. Nat Immunol. 2019;20:163-72.
4. Cheng S, Li Z, Gao R, Xing B, Gao Y, Yang Y, et al. A pan-cancer single-cell transcriptional atlas of tumor infiltrating myeloid cells. Cell. 2021;184:792-809 e23.
5. Tang F, Li J, Qi L, Liu D, Bo Y, Qin S, et al. A pan-cancer single-cell panorama of human natural killer cells. Cell. 2023;186:4235-51 e20.
6. Subramanian A, Tamayo P, Mootha VK, Mukherjee S, Ebert BL, Gillette MA, et al. Gene set enrichment analysis: a knowledge-based approach for interpreting genome-wide expression profiles. Proceedings of the National Academy of Sciences of the United States of America. 2005;102:15545-50.
7. Newman AM, Steen CB, Liu CL, Gentles AJ, Chaudhuri AA, Scherer F, et al. Determining cell type abundance and expression from bulk tissues with digital cytometry. Nat Biotechnol. 2019;37:773-82.
8. Steen CB, Liu CL, Alizadeh AA, Newman AM. Profiling Cell Type Abundance and Expression in Bulk Tissues with CIBERSORTx. Methods Mol Biol. 2020;2117:135-57.
9. Jin S, Plikus MV, Nie Q. CellChat for systematic analysis of cell-cell communication from single-cell transcriptomics. Nat Protoc. 2024, 10.1038/s41596-024-01045-4
10. Chen X, Zhang L, Jin Q, Lu X, Lei J, Peng Q, et al. The clinical features and prognoses of anti-MDA5 and anti-aminoacyl-tRNA synthetase antibody double-positive dermatomyositis patients. Front Immunol. 2022;13:987841.
11. Yang H, Zhang L, Tian X, Li W, Liu Q, Peng Q, et al. Distinct phenotype and prognosis of immune-mediated necrotizing myopathy based on clinical-serological-pathological classification. Rheumatology. 2024, 10.1093/rheumatology/keae361

Supplementary Figures

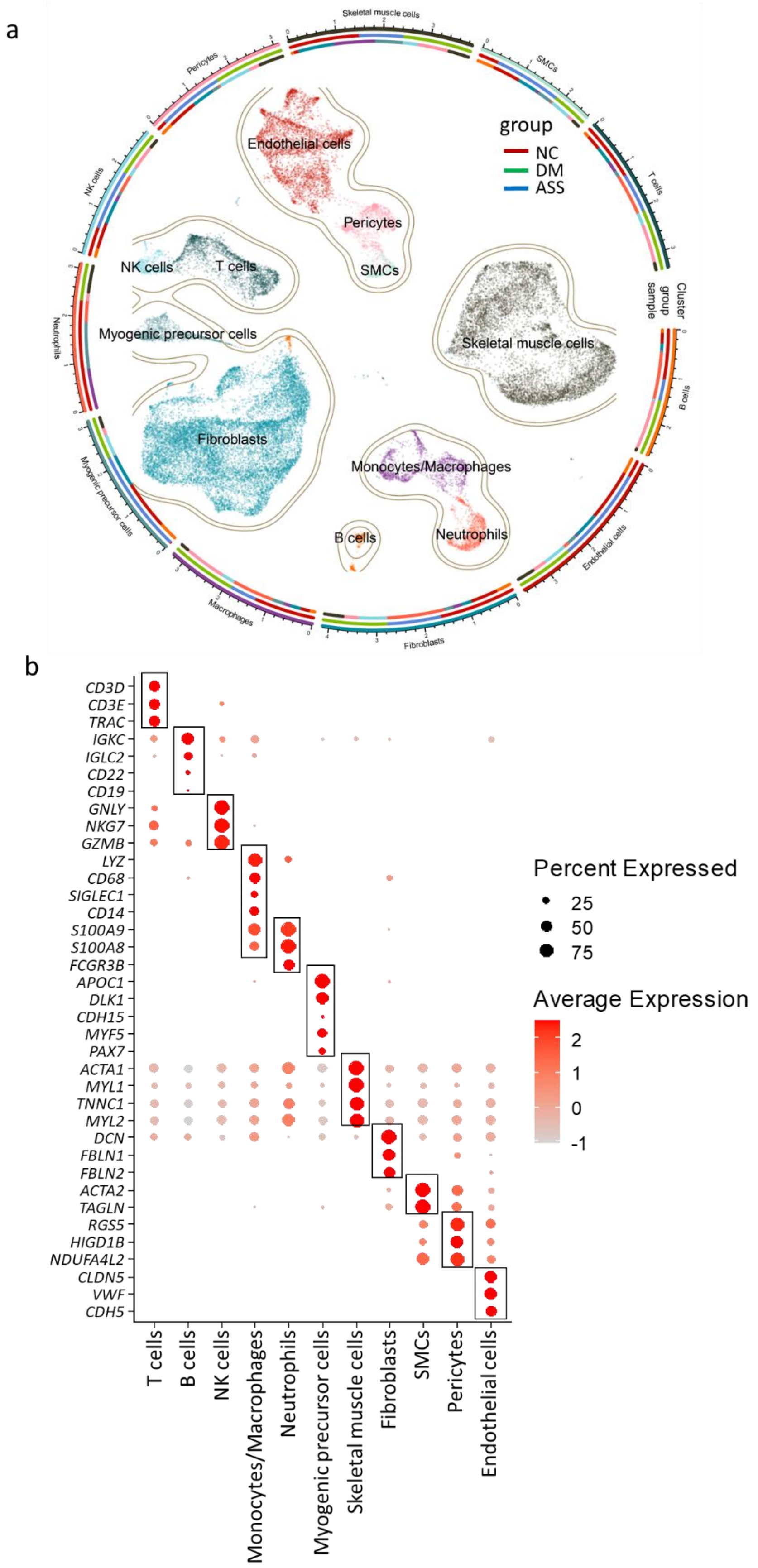

Supplementary Figure 1: Single-cell atlas of cell types in idiopathic inflammatory myopathies (IIMs) muscle tissues.

(a) A total of 34,972 high-quality cells from 3 normal control (NC), 3 dermatomyositis (DM), and 3 anti-synthetase syndrome (ASS) muscle tissues are projected using a uniform manifold approximation and projection (UMAP) plot. Colors indicate contour curves that outline the major cell types and cluster boundaries. The axis outside the circular plot depicts the log scale of the total cell number for each cell class. The three colored tracks (from outside to inside) indicate the cluster (colored as in the central UMAP), group, and sample. The legend shows the group/colors for the group track. (b) Dot plot showing the marker genes characterizing each main cluster, with selected genes listed. Dot size corresponds to the percentage of cells in a cluster expressing the gene, and dot color corresponds to the average gene expression level in the cluster.

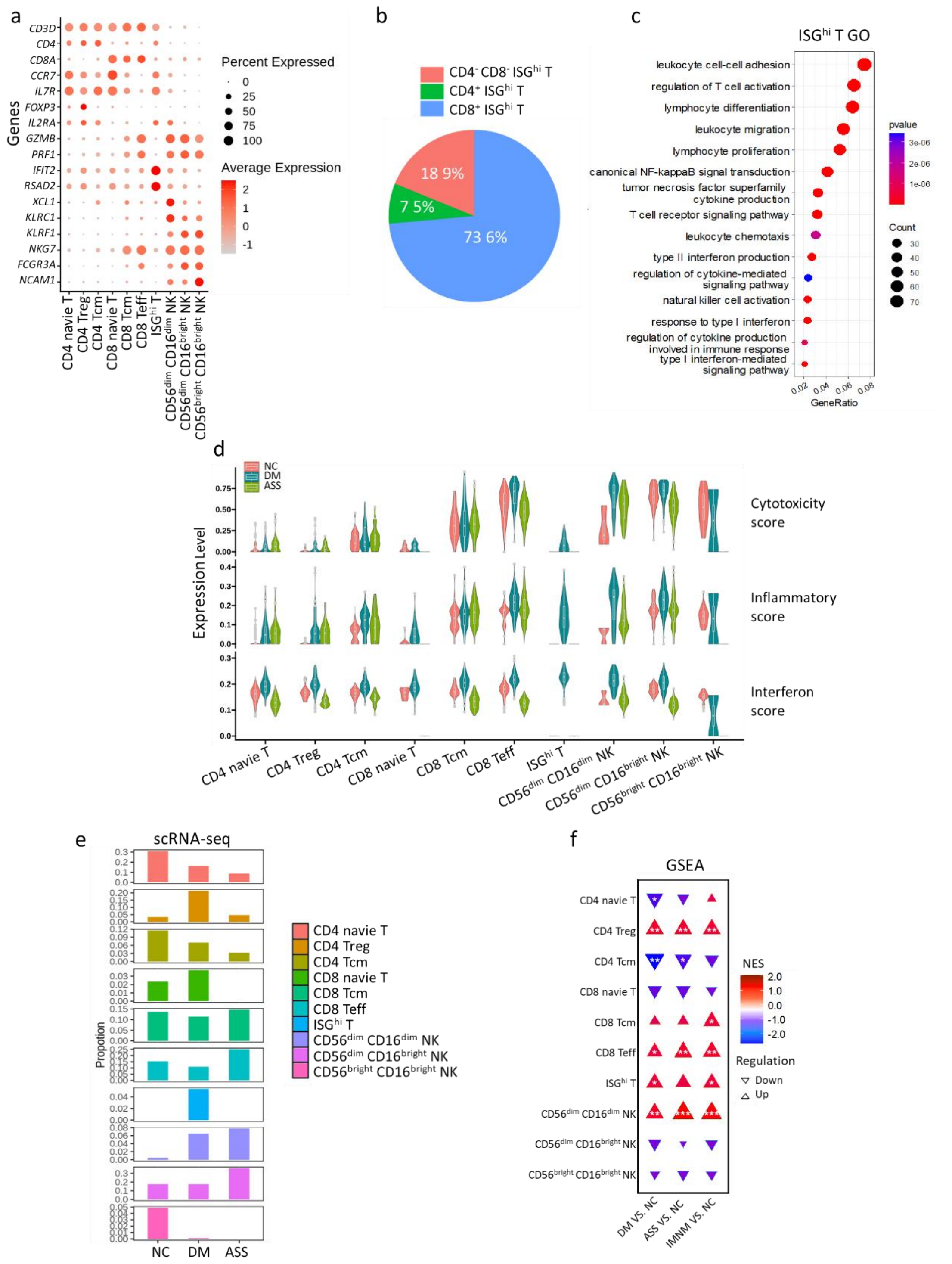

Supplementary Figure 2: Characterization and functional profiling of T and NK cell subpopulations in IIMs.

(a) Dot plot showing the marker genes characterizing each T and NK cell subpopulation, with selected genes listed. Dot size corresponds to the percentage of cells in a cluster expressing the gene, and dot color corresponds to the average gene expression level in the cluster. (b) The percentage of $CD4^+$ and $CD8^+$ ISG^{hi} T cells in the scRNA-seq data from 6 IIMs and 3 NC. (c) Gene Ontology (GO) enrichment analysis was performed on the top marker genes in ISG^{hi} T cells. (d) Violin plots

illustrate cytotoxicity, inflammation, and interferon scores in lymphocyte subpopulations across IIMs subtypes. (e) Percentage of lymphocyte subpopulations in the DM, ASS and NC groups from scRNA-seq data. (f) Gene set enrichment analysis (GSEA) plot of the lymphocyte subpopulations gene sets in the IIMs compared to HC. The colors and size of the dots represent the normalized enrichment score (NES). *$p < 0.05$, **$p < 0.01$, ***$p < 0.001$.

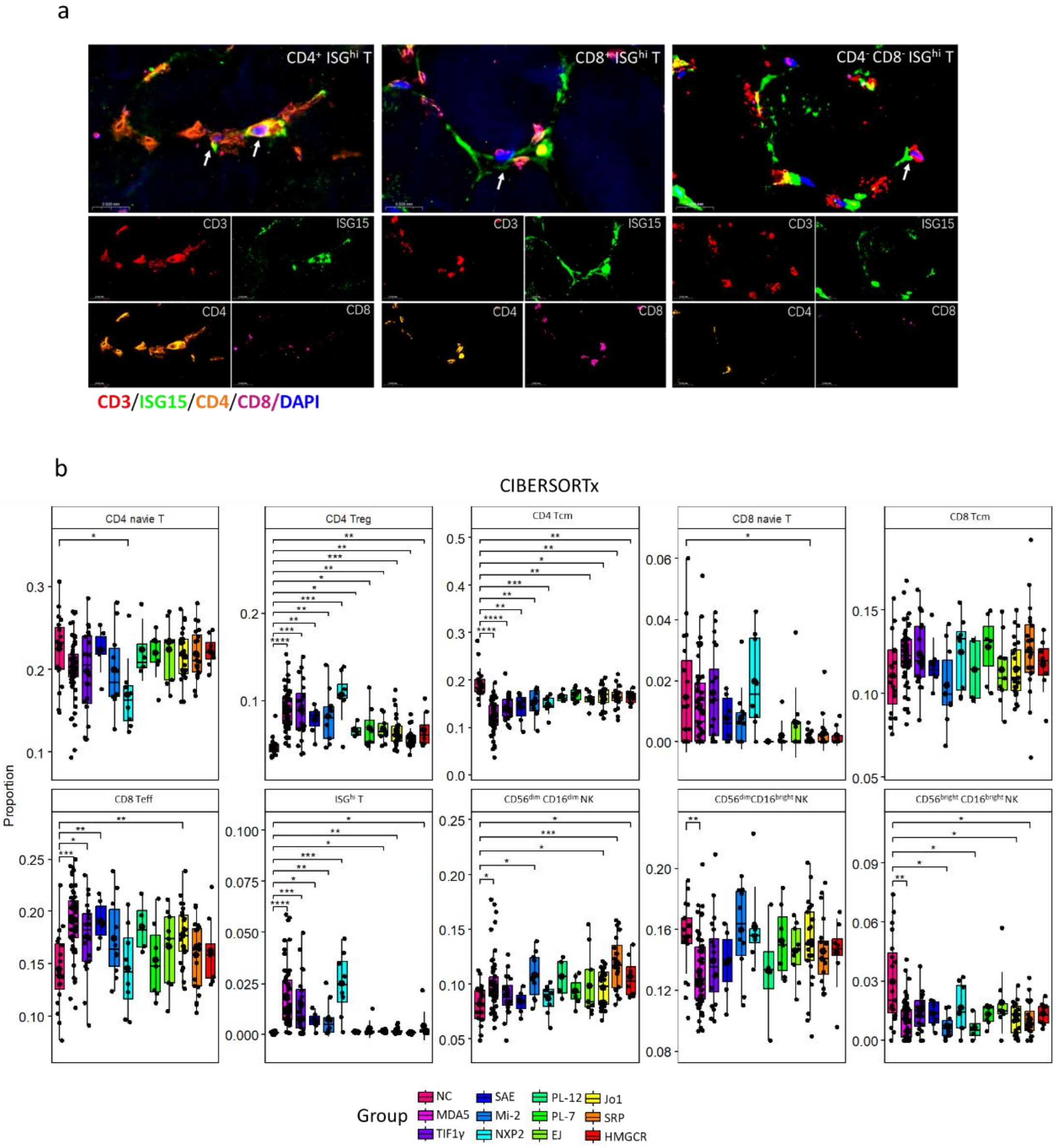

Supplementary Figure 3: Distribution of lymphocyte subpopulations in muscle tissues across IIMs antibody subgroups by CIBERSORTx.

(a) Immunofluorescence identified $CD4^+$, $CD8^+$, and double-negative ($CD4^-CD8^-$) ISG^{hi} T cells in DM muscle tissues. (b) Relative proportions of individual lymphocyte subpopulations in the muscle tissues of IIMs antibody subpopulations and NC as determined by CIBERSORTx analysis. *$p < 0.05$, **$p < 0.01$, ***$p < 0.001$, ****$p < 0.0001$ by unpaired t-test (for normally distributed data) or Wilcoxon test (for non-normally distributed data).

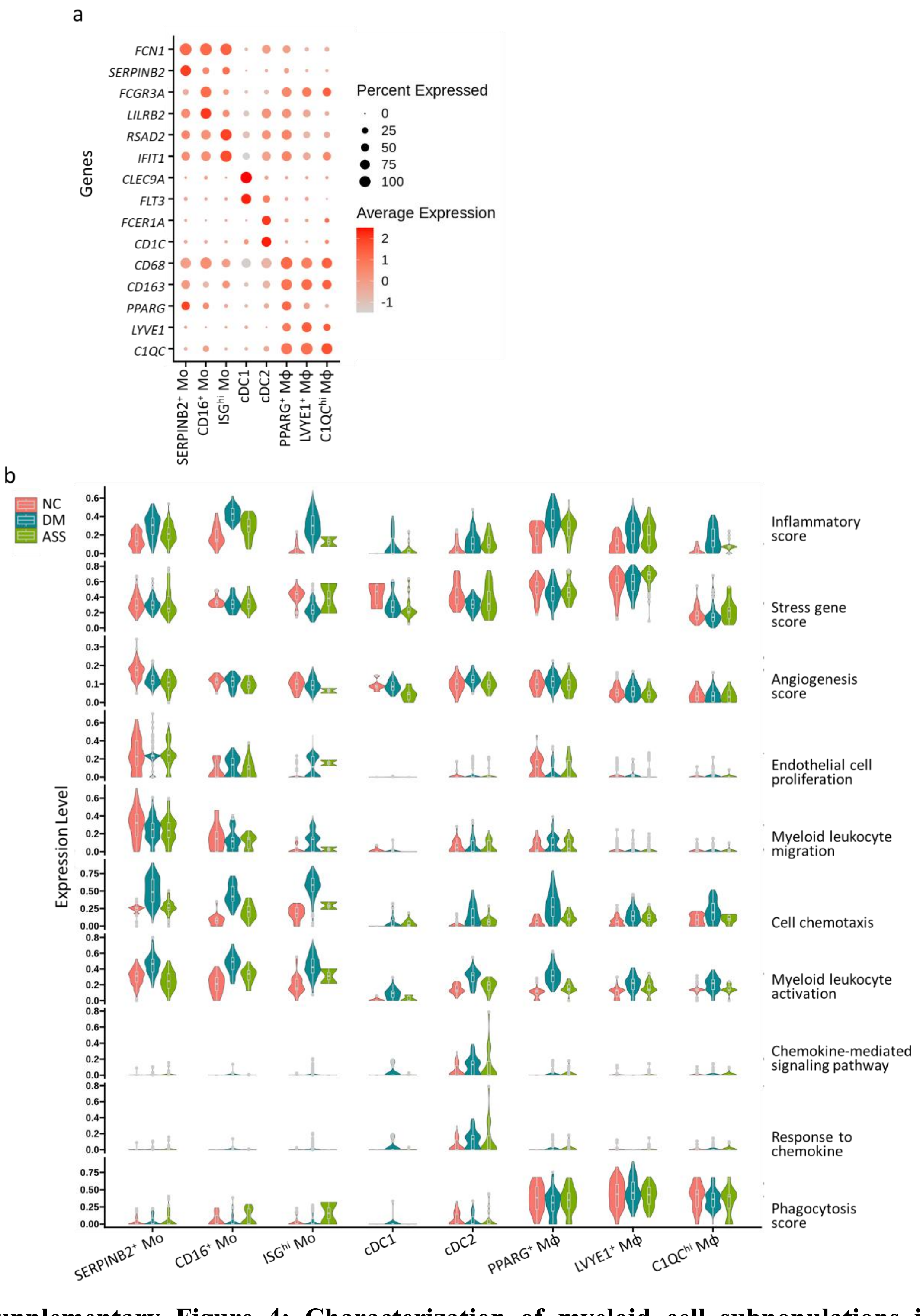

Supplementary Figure 4: Characterization of myeloid cell subpopulations in IIMs.

(a) Dot plot illustrating marker genes that characterize each myeloid cell subpopulation, with selected genes listed. Dot size indicates the percentage of cells in a cluster expressing the gene, and dot color represents the average gene expression level in the cluster. (b) Violin plots depicting the activity of key pathways in myeloid cell subpopulations across different IIMs subtypes.

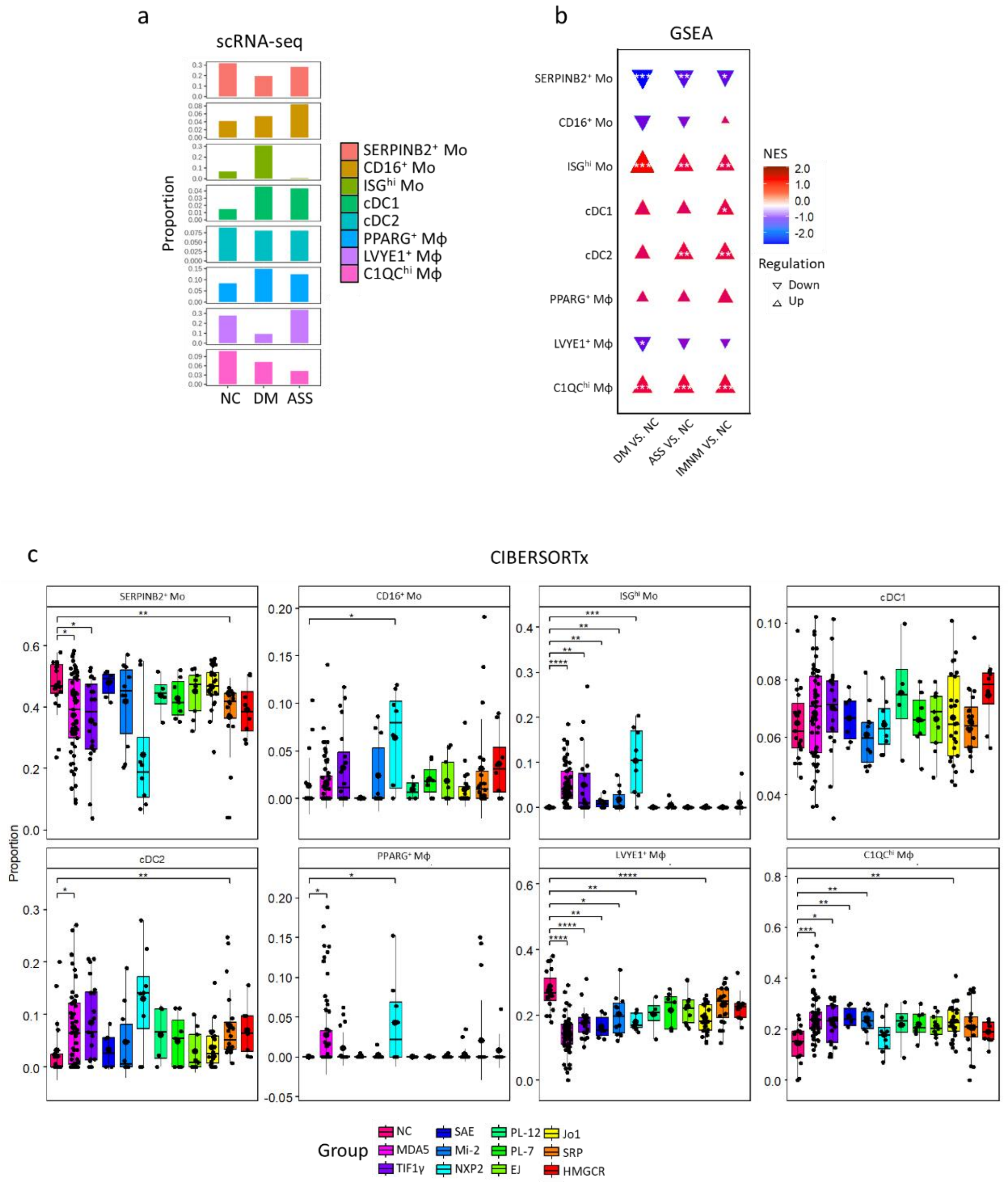

Supplementary Figure 5: Myeloid cell subpopulation analysis in muscle tissues of IIMs antibody subgroups using CIBERSORTx.

(a) Percentage of myeloid cell subpopulations among DM, ASS and NC groups from scRNA-seq data. (b) GSEA plot depicting enrichment of gene sets specific to myeloid cell subpopulations in IIMs compared to HC. The colors and size of the dots represent the NES. **p* < 0.05, ***p* < 0.01, ****p* < 0.001. (c) A comparison of relative proportions of individual myeloid cell subpopulations in muscle tissues among IIMs antibody subgroups and NC was analyzed using CIBERSORTx. Statistical significance was indicated by **p* < 0.05, ***p* < 0.01, ****p* < 0.001, *****p* < 0.0001, determined by unpaired t-test (for normally distributed data) or Wilcoxon test (for non-normally distributed data).

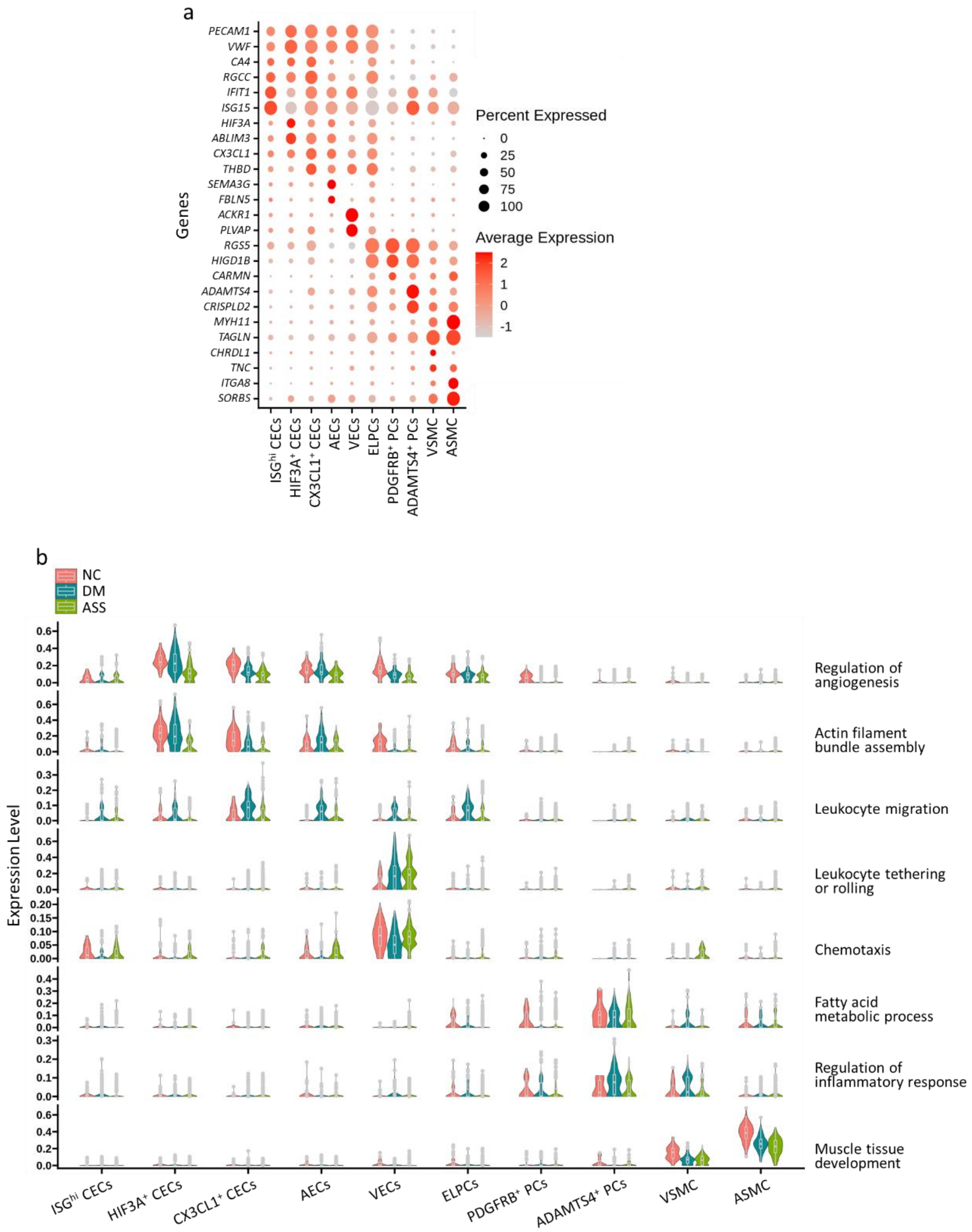

Supplementary Figure 6: Molecular and functional profiling of vascular-related cell subpopulations in IIMs.

(a) Dot plot depicting marker genes characterizing each vascular-related cell subpopulation, with selected genes listed. Dot size indicates the percentage of cells in a cluster expressing the gene, and dot color corresponds to the average gene expression level in the cluster. (b) Violin plots illustrating the activity of key pathways in vascular-related cell subpopulations across different IIMs subtypes.

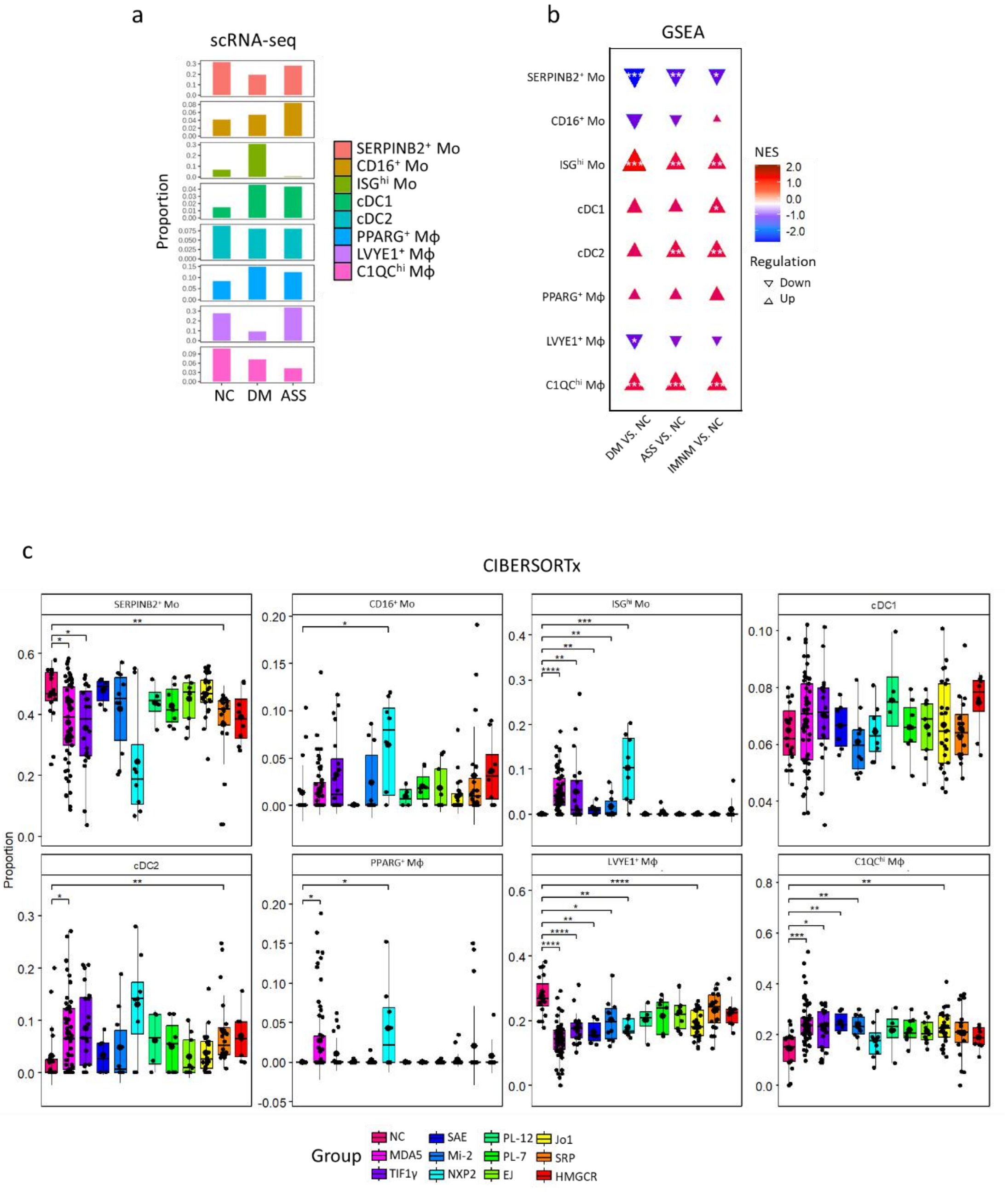

Supplementary Figure 7: Vascular-related cell subpopulation profiling in muscle tissues of IIMs antibody subgroups by CIBERSORTx.

(a) Distribution of vascular-related cell subpopulations among DM, ASS and NC groups from scRNA-seq data. (b) GSEA plot depicting enrichment of gene sets specific to vascular-related cell subpopulations in IIMs compared to NC. The colors and size of the dots represent the NES. *$p < 0.05$, **$p < 0.01$, ***$p < 0.001$. (c) Comparative analysis of the relative proportions of individual vascular-related cell subpopulations in muscle tissues among IIMs antibody subgroups and NC, analyzed using CIBERSORTx. Significance levels were indicated by *$p < 0.05$, **$p < 0.01$, ***$p < 0.001$, ****$p < 0.0001$, determined by unpaired t-test (for normally distributed data) or Wilcoxon test (for non-normally distributed data).

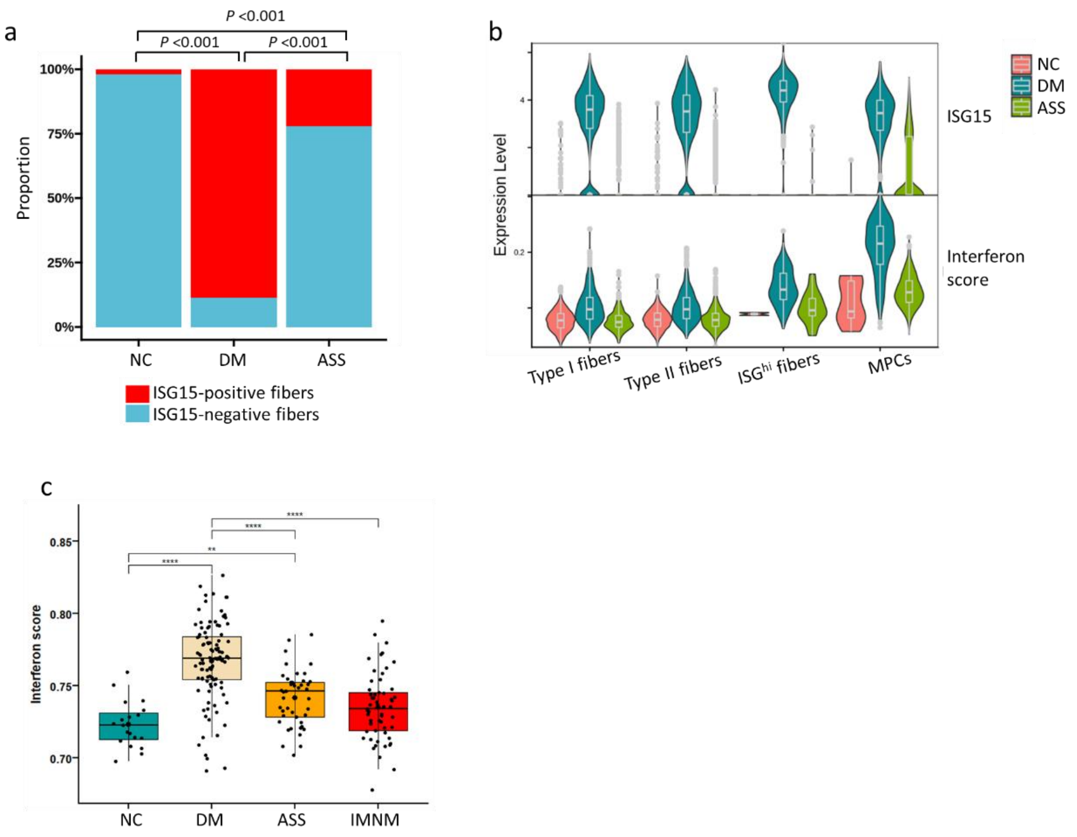

Supplementary Figure 8: Distribution of ISG15-positive muscle fibers and interferon scores across IIMs subtypes.

(a) Proportion of ISG15-positive muscle fibers in DM, ASS, and NC groups based on scRNA-seq data. (b) Violin plots showing ISG15 expression and interferon scores in muscle fibers across IIMs subtypes. (c) Box plot illustrating interferon scores derived from bulk RNA-seq data across different IIMs subtypes. **p < 0.01, ****p < 0.0001, determined by Wilcoxon test.

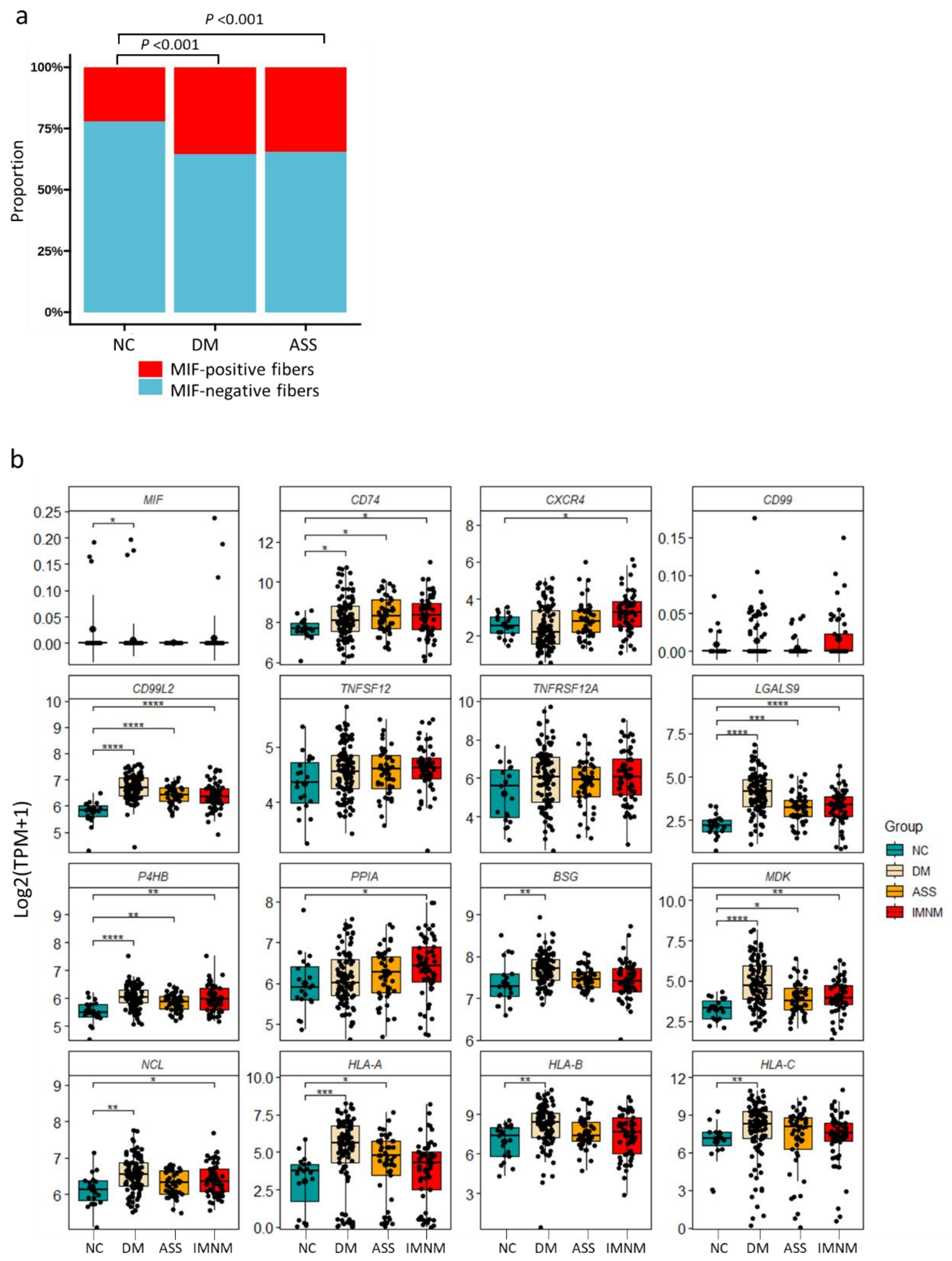

Supplementary Figure 9: MIF-positive muscle fibers and ligand-receptor genes expression across IIMs subtypes.

(a) Proportion of MIF-positive muscle fibers in DM, ASS, and NC groups based on scRNA-seq data. (b) Expression levels of ligand and receptor genes in muscle tissues from IIMs patients. *p < 0.05, **p < 0.01, ***p < 0.001, ****p < 0.0001, determined by unpaired t-test (for normally distributed data) or Wilcoxon test (for non-normally distributed data). TPM: transcripts per kilobase million.

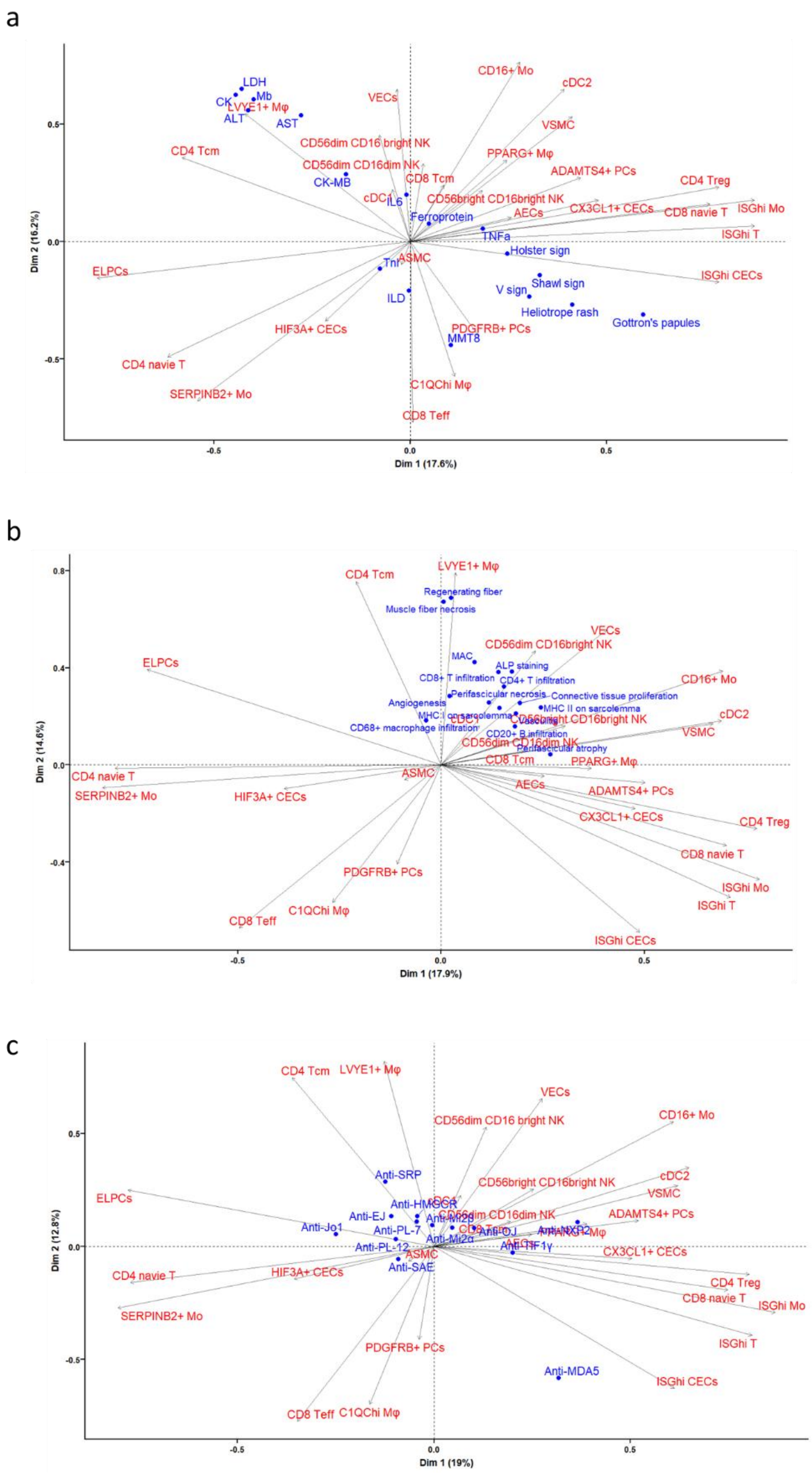

Supplementary Figure 10: Correlations between cell subpopulations and clinical indices, histopathological features, and MSAs.

PCA plots illustrating the relationships between immune and vascular-related cell subpopulations and (a) clinical indices, (b) histopathological features, and (c) MSAs. Immune and vascular-related cell subpopulations are shown in red, while clinical indices, histopathological features, and MSAs are shown in blue.

Supplementary Table 1: Comparison of clinical manifestations and laboratory data among IIMs and NC for Bulk RNA sequencing.

General information	DM	ASS	IMNM	NC	P
Total number	121	45	37	19	-
Age at onset(year)	49.68±11.78	49.23±11.39	49.24±12.37	47.63±15.49	0.992500
Male/female	39/82	14/31	11/26	7/12	0.961909
Disease duration (month)	3.5(2,8)	4.00 (2.00, 9.5)	4(3,8)	-	0.558112
ILD	70	36	15	-	0.001007
Ferroprotein	516.4(200.75,971.4)	476.9(180.4,1067)	388.9(207.8,898.1)	-	0.820377
Heliotrope rash	57	0	0	-	0.000000
Gottron's papules	77	0	0	-	0.000000
V sign	38	0	0	-	0.000000
Shawl sign	28	0	0	-	0.000002
Holster sign	14	0	0	-	0.003259
MMT8	80(69,80)	80.00 (71.50, 80.00)	66(48,74)	-	0.000014
ALT (U/L)	40.9(23.4,78.1)	68.85 (31.15, 159.65)	177.5(87.4,269.5)	-	0.000000
AST (U/L)	51.1(38.5,97.9)	70.40 (47.78, 145.28)	178.3(102.5,287)	-	0.000000
LDH (U/L)	356(299,508)	507.00 (358.80, 797.00)	943(680,1324)	-	0.000000
CK (U/L)	181.8(75,795.2)	1259.50 (558.30, 3832.45)	4879(2713.6,5942.8)	-	0.000000
CK-MB (U/L)	21(14.7,39)	54.6(26.2,98.7)	254.8(118.6,391)	-	0.000000
Mb	81(37.4,352)	532.5(216.1,1200.5)	1588.2(652.7,2535.9)	-	0.000000
TnI	0.011(0.0055,0.025)	0.04(0.0225,0.106)	0.068(0.01675,0.1645)	-	0.000414
TNFα (pg/ml)	11.9(6.545,16.15)	18.60 (12.70, 20.85)	11.6(8,14.525)	-	0.002413
IL6 (pg/ml)	4.605(2.26,11.275)	9.31 (4.33, 15.90)	5.025(2.985,11.825)	-	0.124801
Muscle fiber necrosis	48	32	37	-	0.000000
Mild necrosis	23	14	6	-	-
Severe necrosis	25	18	31	-	-
Perifascicular necrosis	8	12	1	-	0.000515
Perifascicular atrophy	23	4	0	-	0.003177
Vascultis	23	13	6	-	0.295292
Angiogenesis	3	3	7	-	0.002682
Connective tissue proliferation	85	31	31	-	0.098933
Regenerating fiber	41	28	33	-	0.000000
ALP staining	69	28	28	-	0.121665
MAC	36	11	23	-	0.000545
MHC-I expression on sarcolemma	87	32	25	-	0.915945
MHC-II expression on sarcolemma	53	14	11	-	0.264755
CD4+ T cell infiltration	53	19	22	-	0.176634
CD8+ T cell infiltration	40	24	18	-	0.053627
CD20+ B cell infiltration	18	4	2	-	0.271633
CD68+ macrophage infiltration	72	30	27	-	0.317109
MSAs	MDA5+: 57	Jo-1+: 25	SRP+: 20	-	-
	Mi2+: 10	PL-7+: 7	HMGCR+: 8	-	-
	SAE+: 6	EJ+: 9	None:9	-	-
	NXP-2+: 8	PL-12+: 4	-	-	-
	TIF1γ+: 20	-	-	-	-
	None: 20	-	-	-	-

Supplementary Data 1: List of marker genes used for GSEA.
